# Harnessing the Photoperformance of *N*‐Methyl‐Quinolinone for Gated Photo‐Driven Cyclability and Reversible Photoligation

**DOI:** 10.1002/marc.202400474

**Published:** 2024-08-03

**Authors:** Moritz Streicher, Claas‐Hendrik Stamp, Marco Dante Kluth, Alexander Ripp, Céline Calvino

**Affiliations:** ^1^ Cluster of Excellence livMatS FIT‐Freiburg Center for Interactive Materials and Bioinspired Technologies University of Freiburg im Breisgau Georges‐Köhler‐Allee 105 D‐79110 Freiburg Germany; ^2^ Department of Microsystems Engineering (IMTEK) University of Freiburg im Breisgau Georges‐Köhler‐Allee 102 D‐79110 Freiburg Germany

**Keywords:** cyclability, photocycloaddition, quinolinone, reversible photoligation, reversion

## Abstract

[2π + 2π]‐photocycloadditions and their ability to trigger controlled and reversible photoligation through disparate wavelengths provide an attractive platform to unlock advanced functionalities in soft materials. Yet, among the limited amount of functional motifs enabling reversible photoreactions, cyclability is often overlooked due to poor reaction yield and orthogonality. In this study, the advantageous photocharacteristics of the previously underexplored *N*‐methyl‐quinolinone photoresponsive motif are leveraged to create a covalent gated system, enabling controlled formation and cleavage of covalent bonds on demand. A systematic evaluation of individual cycloadditions and reversions on the molecular scale, including reaction rates, conversions, and photoproducts, allows identification of the required conditions for generating controlled photoreactions with a remarkable degree of cyclability; while, maintaining high reaction yields. Ultimately, these controlled and cyclable reactions are translated to a macromolecular scale, showcasing a comparable performance in initiating reversible photoligation, as observed at the molecular level. In addition, it is also shown that this progressive methodology can be leveraged to gain a comprehensive understanding of cyclability and clarify the factors contributing to its decreasing yield. Overall, unlocking the potential of quinolinone derivatives through this step‐by‐step approach lays the foundation for the development of highly controlled and responsive polymer materials with unprecedented potential.

## Graphical abstract

This study explores [2π + 2π]‐photocycloadditions of *N*‐methyl‐quinolinone responsive motifs for controlled and reversible photoligation across disparate wavelengths. Systematic molecular‐scale evaluations reveal optimal conditions, achieving remarkable cyclability and stability. Translating these findings to a macromolecular scale demonstrates comparable performance in reversible photoligation. This progressive methodology provides comprehensive insights into cyclability, shedding light on factors influencing its characteristics.

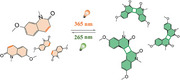

## 1 Introduction

The concept of wavelength‐gated covalent reactions, enabling the selective formation and cleavage of covalent bonds through distinct irradiation wavelengths, holds great potential for unlocking advanced functionalities in soft matter materials.^[^ ^1^, ^2^ ^]^ Such a precise manipulation of molecular reactions has been already harnessed in various reports to achieve remote control over macromolecular architectures, resulting in a diverse range of functionalities such as actuation,^[^ ^3^, ^4^ ^]^ alterations in mechanical properties,^[^ ^5^, ^6^ ^]^ and drug delivery,^[^ ^7^, ^8^ ^]^ alongside other applications.^[^ ^9^, ^10^ ^]^

Among the different reversible light‐mediated reactions, [2π + 2π]‐cycloadditions have emerged as a compelling example of catalyst‐free reactions that can reversibly form and break covalent bonds on demand and under mild conditions.^[^ ^10^ ^]^ These reactions initiate with the excitation of conjugated alkenes within a specific wavelength range, leading to the formation of new *σ*‐bonds through a cyclic transition state, ultimately resulting in the formation of a cyclobutane moiety.^[^ ^11^ ^]^ Subsequent exposure to distinct irradiation can undo this transformation, reverting the structure back to the original alkenes.^[^ ^12^ ^]^ Note that [2π + 2π]‐cycloaddition is not an inherent characteristic of all alkenes and typically requires an extended system of conjugation to enable the photochemical reaction. Similarly, not all cyclobutanes can symmetrically reverse into the original alkenes.^[^ ^13^, ^14^ ^]^ Thus, only a limited number of chemical motifs, including derivatives of coumarin,^[^ ^15^ ^]^ stilbene,^[^ ^16^ ^]^ maleimide,^[^ ^17^ ^]^ thymine,^[^ ^18^ ^]^ and cinnamic acid,^[^ ^19^ ^]^ have been reported to enable reversible reactions.^[^ ^20^ ^]^ When incorporated into polymers, these photoresponsive motifs, capable of intermolecular coupling of macromolecular chains, have proven successful in inducing diverse property changes in bulk polymers, such as being used as crosslinking units to enhance stiffness in hydrogels and to improve repairing capabilities in coatings.^[^ ^21^, ^22^, ^23^ ^]^ These systems have also found in situ and in vitro applications in biological processes,^[^ ^24^, ^25^ ^]^ further expanding their scope and potential impact in various technological fields. However, while holding great promise for macroscopic modulations, only a few of these motifs exhibit true gated characteristics, thereby limiting full control of their responsive potential, and consequently, of the materials' functionality.^[^ ^26^, ^27^ ^]^ This limitation is typically attributed to the relatively low yield (<80%) of the photoreactions, coupled with the occurrence of a so‐called photostationary state—a phenomenon characterized by the equilibrium of both the forward and reverse photoprocesses under continuous irradiation, typically resulting from the absorption spectra overlap between the alkene adducts and the cyclobutane products.^[^ ^28^, ^29^, ^30^ ^]^ Reaction yields, including reversions, have been enhanced catalytically by employing triplet sensitizers^[^ ^31^ ^]^ and Lewis acids^[^ ^32^ ^]^ or through the use of template supramolecular systems.^[^ ^33^ ^]^ Alternatively, structural modification of the existing responsive motifs has also been exploited to enhance the reaction yields^[^ ^34^ ^]^ and simultaneously minimize the photostationary state by maximizing the monomer/dimer absorption decoupling.^[^ ^21^, ^26^, ^35^ ^]^

Complementarily, Barner–Kowollik and coworkers have emphasized the importance of conducting a wavelength dependency analysis to identify the most effective irradiation wavelengths for triggering efficient and disparate photoreaction pathways (i.e., cycloaddition and cycloreversion).^[^ ^36^ ^]^ Using a tunable laser, the wavelength‐dependent conversion of photochemical component could be mapped in a so‐called action plots. As a result of this mapping, Marschner et al. enabled to identify an entirely *λ*‐orthogonal photoreversible polymer ligation of poly(ethylene glycol) derivative upon reversible dimerization of styrylpyrene at disparate wavelengths of 435 and 330 nm.^[^ ^26^ ^]^ In another example reported by Irshadeen et al., the action plots enabled to identify a reactivity red‐shifted by 25 nm relative to the absorption maximum at 415 nm of a photoreactive pyrene chalcon moiety; and thus, to enable a green light LED activation, rendering these systems viable for biological applications.^[^ ^37^ ^]^ Despite these notable advances, efforts to use photocycloadditions as a foundation for reversible ligations have encountered challenges, such as photoreversible polymerizations being limited to templating environments and exhibiting low degrees of polymerization. Consequently, further exploration of effective reversible photochemistries is necessary to fully harness their gated functionality within polymeric systems.^[^ ^38^ ^]^ In this context, the *N*‐methyl‐quinolinone motif has captured our interest owing to its superior photoperformance in comparison to its extensively studied counterpart, the coumarin scaffold. *N*‐methyl‐quinolinones are lactam analogs of coumarins, which have been previously reported to enable reversible photocycloadditions.^[^ ^39^ ^]^ Hampp and coworkers previously demonstrated that *N*‐methyl‐quinolinones outperform their analog coumarins in reaction rates, attributed to a higher quantum yield.^[^ ^39^ ^]^ However, further investigations on the photochemistry of this motif revealed the formation of oxidative side species, specifically bis‐*N*‐methylquinolinone (bis‐NMQ), resulting from the reaction of the anti‐head‐to‐head *N*‐methylquinolinone dimer with oxygen during the cycloaddition.^[^ ^40^ ^]^ This photo‐oxidation process showcased a constrained reversibility range for the respective motif, which had ultimately contributed to its disregard.

**Figure 1:**
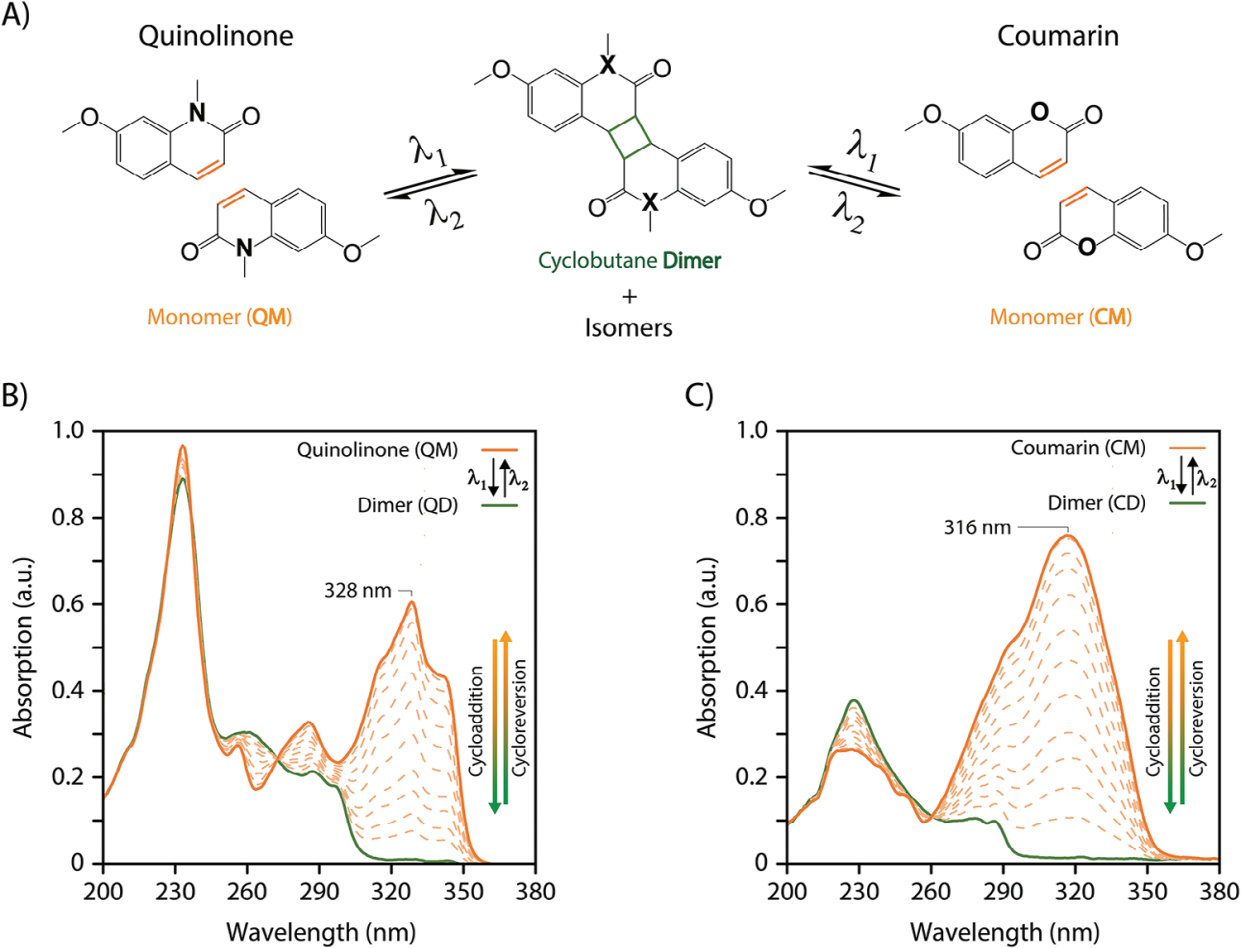
A) Schematic representation of the light‐driven [2π + 2π]‐photocycloaddition and reversion of quinolinone (QM) and coumarin (CM). B) Corresponding UV–vis absorption spectra of the cycloaddition (orange to green) and reversion (green to orange) of QM and C) of CM in acetonitrile at *c* = 5.67 × 10^−5^ mol L^−1^.

Building on these initial observations, we leverage the advantageous photocharacteristics of this *N*‐methyl‐quinolinone in the realm of wavelength‐gated covalent chemistry, aiming to achieve efficient and highly controllable reversible photoligation in polymeric systems. By establishing procedures to avoid the formation of side products, we demonstrate the ability to optimize reaction yield and fully capitalize on the rapid photoprocess. The introduction of systematic studies for individual photoreactions allows us to identify optimal parameters for efficient individual photoprocesses, ultimately unlocking an unprecedented gated functionality upon their combination. In contrast to previous studies conducted in flow reactors,^[^ ^40^ ^]^ this investigation focuses on examining photoreactions in static solutions. Given the complexity of photochemical reactions and their outcomes, which are influenced by multiple parameters, conducting systematic and comprehensive investigations is critical for gaining a thorough understanding and improving control over these reactions. Thus, this study starts by investigating the photoperformances of 7‐methoxy‐1‐methylquinolin‐2(1*H*)‐one (QM), including [2π + 2π]‐cycloaddition and reversion reactions, and draws comparison with its analogue, 7‐methoxycoumarin (CM) (**Figure** **1**A–C).

## 2 Results and Discussion

### 2.1 [2π + 2π]‐Photocycloaddition

**Figure 2:**
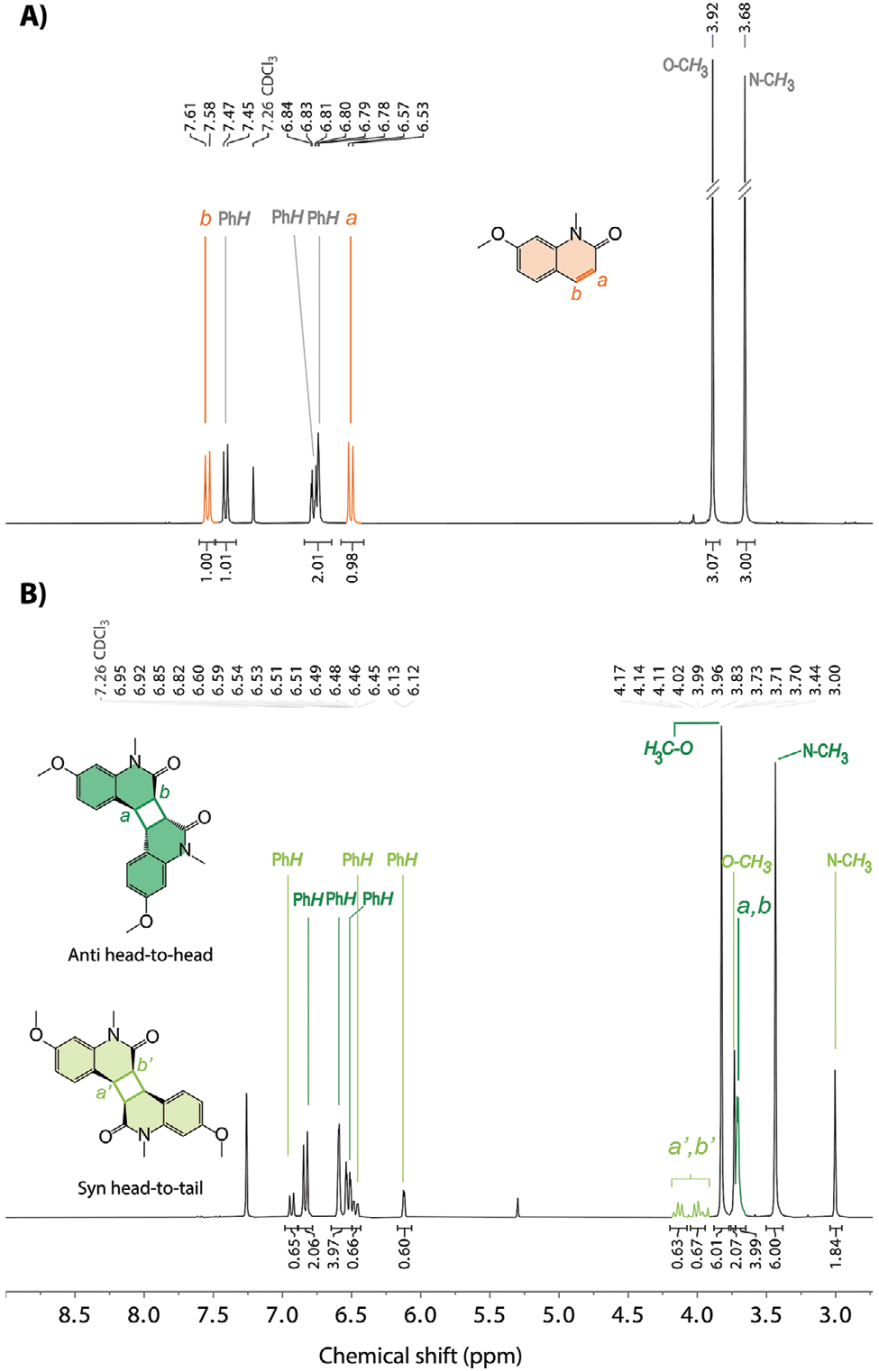
Comparison of the ^1^HNMR spectra (400 MHz, 297.2 K, CDCl_3_) of A) QM and B) the produced dimer stereoisomers, i.e., *aHH* and *sHT*, upon LED irradiation at 365 nm at *c* = 0.57 mol L^−1^.

The synthesis of QM and CM was conducted according to previously reported procedures (see details in the Supporting Information). Following standard protocols for the cyclization of coumarin motifs, typically triggered above 350 nm,^[^ ^20^ ^]^ QM was dissolved in acetonitrile (*c* = 5.28∙10^−5^ mol L^−1^) and irradiated with a LED lamp at *λ* = 365 nm (at a power of 450 mW) for 120 h (see details in the Supporting Information). To prevent any competitive oxidative side reactions reported to occur during the cycloaddition (Figure S1, Supporting Information),^[^ ^40^ ^]^ these irradiations were conducted under oxygen‐free conditions. Note that the formation of side products is one parameter contributing to the decrease in yield, consequently impacting the cyclability performance. In addition, it is worth noting that acetonitrile is a common solvent used for photoreactions due to its photostability and low absorption in the UV range. UV–vis absorption spectra recorded during the irradiation process showed a reduction in the absorption band at 328 nm and the formation of an isosbestic point at 274 nm, suggesting a stoichiometric reaction and the emergence of a new entity (Figure 1B; Figure S2, Supporting Information). Moreover, the absorption spectra showed no evidence of the characteristic photo‐oxidative side product (Figure S2, Supporting Information).^[^ ^40^ ^] 1^H‐NMR spectrum of the produced product displayed various shifted signals in the aromatic region and revealed the appearance of additional magnetic resonances compared to the reference spectra of QM, both implying the formation of multiple photoproducts (**Figure** **2**). Furthermore, the absence of the typical QM signals indicated a full conversion of the reactants (QM) into products. Further analysis of the ^1^H‐NMR, in conjunction with X‐ray diffractometric measurements, led to the identification of stereoisomers of the dimer, specifically the anti Head‐to‐Head (*aHH*) and syn Head‐to‐Tail (*sHT*) photoproducts (Figure 2B; Figure S3, Supporting Information and CCDC 2257859). The correlation between the methyl signals at 3.00 and 3.44 ppm, corresponding to the N─CH_3_ groups of both isomers, allowed for the quantification of the photoproducts. The NMR analysis revealed a predominant presence of the *aHH* product (76%) and a minor formation of the *sHT* isomer (24%). It is noteworthy that previous studies did not report a mixture of isomers, emphasizing the importance of conducting systematic studies for every photoprocess method.^[^ ^40^ ^]^

**Figure 3:**
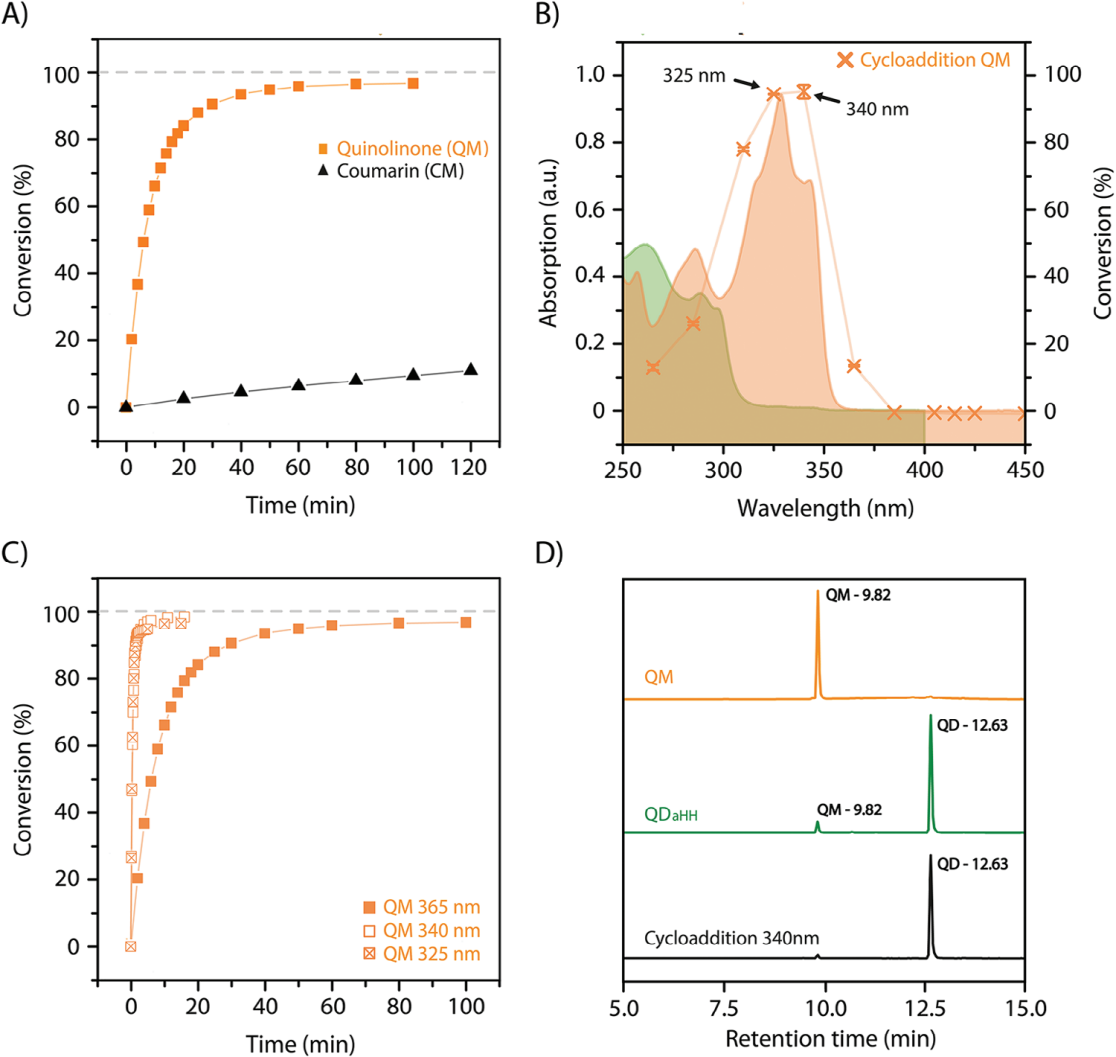
A) Comparison of the photocycloaddition conversions of QM (orange squares) and CM (black triangles) over time. B) Action plot obtained with irradiations of 3.22 × 10^19^ photons (orange crosses) at each wavelength, as well as the UV–vis spectra of QM (orange) and QD (green). C) Comparison of the photocycloaddition conversions of quinolinone under irradiation at 365, 340, and 325 nm. D) Comparison of the HPLC chromatograms of QM (orange), QM after irradiation at 340 nm for 12 min (black), and QD*_aHH_* (dark green). All photoreactions in (A–D) were conducted in acetonitrile solutions at *c* = 10^−5^ mol L^−1^ and irradiated with a LED photoreactor at powers ranging from 22 to 450 mW.

Upon the demonstration of the dimerization reaction, kinetics of the photocycloaddition were conducted to determine the reaction rates and assess the conversion efficiency of the photoreaction. Thus, diluted QM solution in acetonitrile (*c* = 4.7·10^−5^ mol L^−1^) was irradiated for 100 min at 365 nm at a power of 450 mW and the resulting absorption spectra recorded at regular intervals of few minutes. The conversion at each stage was determined by using the molar extinction coefficient of the quinolinone (see details in the Supporting Information). Plots of the calculated conversion as a function of the irradiation time showcased a rapid formation of dimer products within the first 20 min of irradiation, reaching a plateau after 60 min with a maximum conversion of 97% (**Figure** **3**A).

In order to evaluate the photoperformance of the [2π + 2π]‐photocycloaddition reaction of QM, similar investigations were carried out on the irradiation of analogue coumarins CM (Figure S4, Supporting Information). Comparative kinetic studies conducted under oxygen free conditions demonstrated that the cycloaddition reaction rate of QM was two orders of magnitude higher than that of CM (Figure 3A). Furthermore, QM displayed a higher reaction conversion (97%) in contrast to CM (75%), consistent with earlier findings by Hampp and coworkers conducting these reactions in the presence of oxygen.^[^ ^39^ ^]^ While the dimerization process of coumarins may be accelerated under oxygen conditions, these dimerization rates still do not match those of quinolinones under nitrogen (Figure S5, Supporting Information). Finally, consistently with the literature,^[^ ^41^ ^]^ CM also exhibited the formation of two isomers (syn Head‐to‐Tail and anti Head‐to‐Head) compared to QM (Figure S6, Supporting Information).^[^ ^42^ ^]^ These comparative studies demonstrate the superior performance of the dimerization process of quinolinones when conducted under inert conditions, offering an effective (i.e., faster and higher conversions) and highly controllable (i.e., formation of single isomers) dimerization process.

Inspired from earlier reports on wavelength‐by‐wavelength mapping of photochemical reactivity,^[^ ^36^, ^43^ ^]^ a wavelength screening of the photochemical response of the reaction was conducted to identify the optimal photodimerization wavelength for maximizing the conversion of QM into QD (Figure 3B).^[^ ^26^ ^]^ Thus, solutions of QM in acetonitrile were irradiated using a custom‐made photoreactor equipped with LEDs at different wavelengths (265, 285, 310, 325, 340, 365, 385, and 410 nm). All irradiations were carried out at a constant concentration (*c* = 5.67·10^−5^ mol L^−1^) and at a fixed photon count (calculations of the photon count and information about the photoreactor are provided in the Supporting Information). The resulting products were quantified by UV–vis spectroscopic measurements. Interestingly, plots depicting the conversion of QM as a function of the irradiation wavelength revealed a significantly higher conversion at 340 and 325 nm compared to other wavelengths (Figure 3B). Consistently, kinetics performed at these two wavelengths exhibited higher reaction rates and conversions compared to 365 nm (Figure 3C). While one might expect high reactivity for solutions irradiated at 325 nm due to the maximum absorbance of the monomer, these results highlight a small disparity between absorptivity and photochemical reactivity at 340 nm. The latter observed redshifted enhancement of reactivity—already reported for various photochemical reactions^[^ ^44^, ^45^, ^46^ ^]^—still remains an unclear phenomenon; although, some rationalizations have been reported.^[^ ^47^ ^]^ Nonetheless, our findings confirm the significance of performing a wavelength screening for optimizing photoreactions.

Given the reactivity similarities between kinetics conducted at 325 and 340 nm, the most redshifted wavelength was selected to pursue the investigation. Thus, further irradiations at 340 nm (33 mW) were conducted at higher concentrations (*c* = 0.57 mol L^−1^) to investigate the molecular structure of the photoproducts. In this case, analysis of the ^1^H‐NMR spectra revealed the presence of identical *aHH* and *sHT* stereoisomers, with a significant predominance of *aHH*, mirroring the findings from the experiment conducted at 365 nm (Figure S7, Supporting Information). Further analyses revealed the capability to selectively produce a single a*HH* isomer by controlling the reaction time during the initial phase of the plateau. The HPLC chromatogram of the irradiated solutions in the diluted state indicated the sole presence of a*HH* isomers (Figure 3D); thereby, positioning quinolinones as promising candidates for efficient and precisely controlled systems. Overall, these comprehensive investigations unequivocally establish the superior photocharacteristics of quinolinone motifs in inducing [2π + 2π]‐photocycloaddition reactions. The implementation of these established procedures under oxygen‐free conditions, combined with the screening of wavelengths, resulted in a comprehensive approach to achieve high cycloaddition conversion, approaching 100% and substantially increasing the reaction rates. It is worth noting that similar performances have only been achieved with the use of additional oxygen scavenger additives and quenchers (such as ascorbic acid and NaN_3_), which, as a result, hinder the benefits of these catalyst‐free reaction schemes.^[^ ^40^ ^]^

### 2.2 Cycloreversion

The performance of the photo‐mediated cycloreversion of QD was investigated and compared to CD reversion using the same systematic procedure outlined previously. In this case, a wavelength of 265 nm was selected to trigger the reversion of QD, as widely reported for reversing coumarin dimers.^[^ ^48^, ^49^ ^]^ Kinetics were conducted using pure *aHH* dimer (QD*_aHH_*), which was dissolved in acetonitrile (*c* = 3.5 × 10^−5^ mol L^−1^) and irradiated with a LED lamp at *λ* = 265 nm for 5 min under oxygen‐free conditions (details in the Supporting Information). The absorption spectra, collected at regular intervals (10–30 s), displayed a progressive increase of the characteristic absorption band of QM at 328 nm. After 3 min of irradiation, the spectra showed an identical pattern to that of the original QM, suggesting the cleavage of the cyclobutanes (Figure 1A). The recovery of the original monomers QM was confirmed by ^1^H‐NMR spectroscopic analysis of samples irradiated at higher reaction concentrations (*c* = 2.7 × 10^−3^ mol L^−1^) for 18 h. The spectra exhibited a clear regression of the signals previously assigned to the QD; while, the recovery of the CH double bonds at 7.60 and 6.56 ppm, as well as the aromatic and heteroatom methyl N─CH_3_, O─CH_3_ resonances corresponding to QM, was observed simultaneously (Figure 2; Figure S8, Supporting Information).

**Figure 4:**
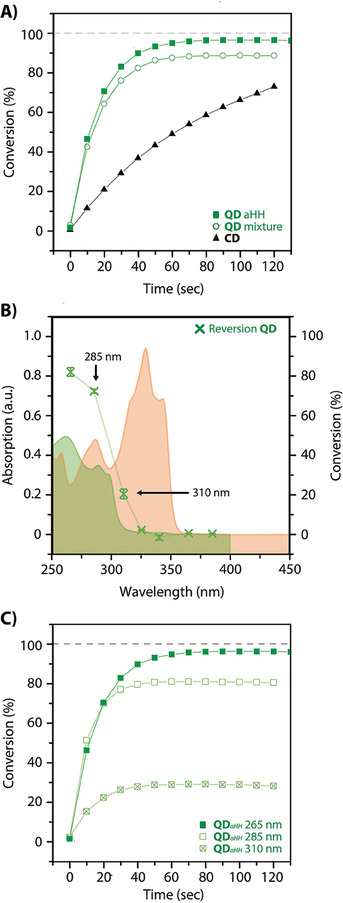
A) Comparison of the photocycloreversion conversions conducted under oxygen‐free conditions for pure QD*_aHH_* (green squares), a mixture of QD*_aHH_* and QD*_sHT_* (circles), and CD (black triangles) over time and upon irradiation at 265 nm. B) Action plot of QD (green crosses) obtained with irradiations of 3.22 × 10^19^ photons at each wavelength, as well as the UV‐vis spectra of QD (green) and QM (orange). C) Comparison of the photocycloaddition conversions of QD upon irradiations at 265, 285, and 310 nm. Photoreactions in (A–C) were carried out in acetonitrile solutions at a concentration of *c* = 10^−5^ mol L^−1^. The samples were irradiated using a LED photoreactor at power levels ranging from 22 to 80 mW.

A comparative study on the reversion reaction in the diluted state of QM and CM, monitored by UV–vis spectroscopy, revealed higher reaction rates and remarkable conversion reaching 96% for QD compared to CD, displaying a notably milder slope (**Figure** **4**A). The irradiation of a mixture of *aHH* and *sHT* dimers in a 97:3 ratio yielded a marginally lower reversion conversion; yet, remaining at a substantial 90%. This observed constrained conversion can be attributed to the equilibrium established between the cycloaddition and reversion, often denoted as the photostationary state. Accordingly, the irradiation of QM at 265 nm has shown the generation of dimers overtime (Figure S9, Supporting Information), suggesting the presence of this pathway in the cycloreversion process. Although theoretically, this equilibrium could contribute to decreasing the conversion of reversion, our experiments demonstrate that this phenomenon remains irrelevant in the overall equilibrium, as shown by the full reversion of QD*_aHH_*. The influence of the dimer mixture on the reversion process; however, remains uncertain; nevertheless, this aspect becomes inconsequential given that the previously established cycloaddition methodology facilitates the controlled formation of the *aHH* isomer.

As previously conducted for the cycloaddition, a wavelength dependency analysis was performed on the cycloreversion of QD*_aHH_*. In this analysis, no wavelength demonstrated better efficiency compared to 265 nm (Figure 4B). Furthermore, while the 285 and 310 nm redshifted wavelengths exhibited some conversions (Figure 4C), their conversion efficiencies were lower (80% and 30%, respectively), ultimately rendering these wavelengths unfavorable for cycling process. These restricted conversions were presumably due to the larger absorption spectra intensity of QM when compared to QD, not allowing the reaction rates to compensate the photostationary state this time. These experiments exemplify again the need to perform wavelength screening to maximize the orthogonality of the photoreactions.

### 2.3 Photocycling

Having optimized both photoreactions (i.e., cycloaddition and cycloreversion) and demonstrating the superior performance of quinolinones in terms of reaction rates, conversions, and controlled formation of isomers, compared to coumarins, this study then shifted its focus exclusively toward examining the cyclability of these photoreactions for quinolinones.

**Figure 5:**
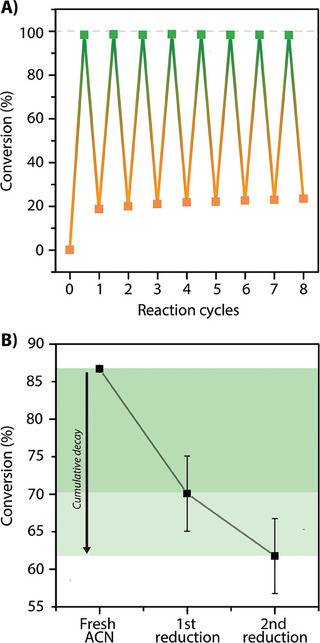
A) Subsequent irradiation cycles of QM conducted at 340 nm for the cycloaddition and at 265 nm for the reversion. The photoreactions were carried out under oxygen‐free conditions at *c* = 10^−5^ mol L^−1^ using a LED photoreactor at power levels ranging from 22 to 450 mW. B) Conversion of QD after one cycle of subsequent cycloaddition and reversion conducted at irradiation wavelengths of 340 and 265 nm (*c* = 10^−5^ mol L^−1^), respectively, as function of the cumulated residues in acetonitrile. The residues were cumulated by evaporating 1 L (first data point) to 50 mL (second data point), and further reduced to 12 mL for the third data point.

Based on previous results and to maximize the orthogonality of the process, 340 and 265 nm were chosen to trigger cycloaddition and cycloreversion, respectively, under oxygen‐free conditions. Thus, the irradiations were conducted in a one‐pot reaction under inert conditions following the previously described procedures (*c* = 5.0 × 10^−5^ mol L^−1^), and the conversions over the reaction time were tracked by UV–vis spectroscopic measurements (**Figure** **5**A). In accordance with the previous examination of the individual processes, the initial cycloaddition exhibited high conversion of ≈98%, within a brief period (≈ 3 min). However, in an unexpected turn, the reversion process achieved merely a limited conversion of 80%, which contrasts with the usual trend of reversion processes displaying conversions exceeding 90%. More interestingly, a remarkably high level of cyclability with robust stability was achieved by alternating the cycloaddition and reversion processes through a total of eight cycles (Figure 5A)—a performance hardly demonstrated in solution for these types of responsive systems, to the best of our knowledge.

Still, this initial discrepancy, which has also been noted in previous reports,^[^ ^26^ ^]^ remained the only restriction toward a fully potent cyclable system. Previous reports have attributed this phenomenon to an interplay between the photostationary state and a photo‐bleaching process that asymmetrically cleaves the chemical motifs.^[^ ^26^ ^]^ However, our progressive approach, studying the individual photoreactions, has already demonstrated the irrelevance of the photostationary state for these quinolinone reactions when triggered at these particular wavelengths. Interestingly, upon examining the HPLC chromatogram after the first irradiation cycle (Figure 5B), an expected rise in the signal corresponding to QM was evident. However, surprisingly, only a partial decrease in the signal previously attributed to the *aHH* isomer (Figure 3D; Figure S10, Supporting Information) was observed, suggesting that a portion (≈20%) of the dimer remained unreacted. Note that no other products were observed. Subsequent repetitions of this initial cycle process were conducted to isolate the photoproducts in a larger quantity and enable the characterization of their structure through NMR spectroscopy. While the compound assigned to the retention time of 9.82 min revealed a spectrum corresponding to QM, the second compound at retention 12.63 min (Figure S10, Supporting Information) decomposed once mixed with the solvent, indicating the formation of unstable dimer side products during this initial photoprocess cycles (Figure S11, Supporting Information). Further investigations revealed that this discrepancy was associated with solvent residues. While attempts to purify the solvent proved unsuccessful, the accumulation of these residues by reducing a large amount of solvent (1 L) to few mL showed a correlated loss of reversion in the first cycle of the photoprocess. Alternatively, replacing the irradiated solvent after the first cycle with fresh solvent for the second cycle led to an additional loss of ≈10%, which further increased upon repeating this procedure (Figure S12, Supporting Information). These combined experiments allowed us to attribute the initial loss of reversion conversion to solvent residues, presumably present in a catalytic amount, and ultimately, confirming the exceptional performance of quinolinones in terms of cyclability.

### 2.4 Reversible Photoligation

**Figure 6:**
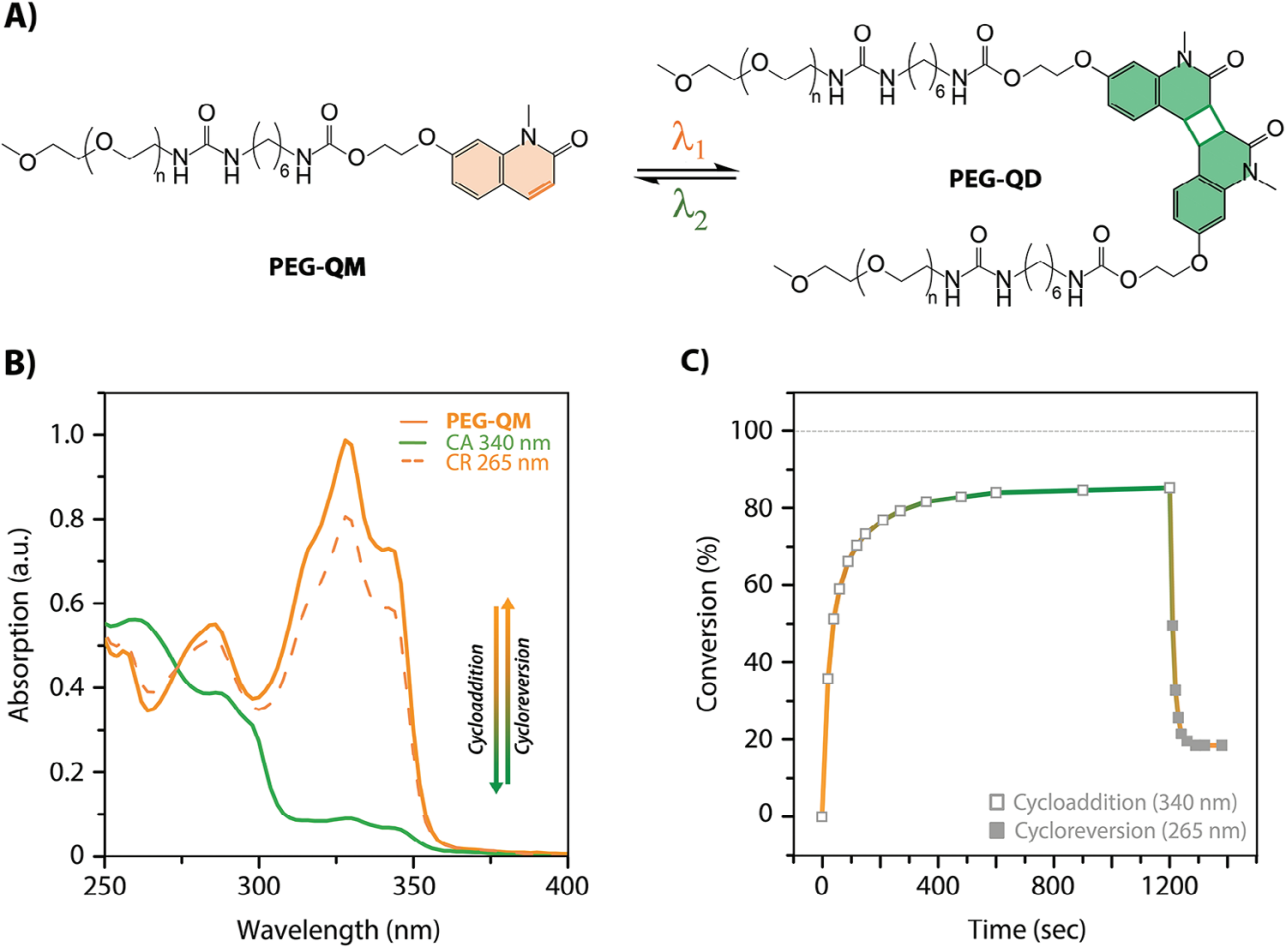
A) Schematic representation of the reversible photoligation of quinolinone terminal polyethylene glycol (PEG‐QM) via [2π + 2π]‐cycloaddition and reversion. B) UV–vis absorption spectra of the cycloaddition (orange to green) and subsequent reversion (green to dashed orange) of PEG‐QM. C) Corresponding reaction conversions. All photoreactions in (B–C) were conducted in acetonitrile solutions at *c* = 10^−5^ mol L^−1^ under oxygen‐free conditions and irradiated with a LED photoreactor at powers ranging from 22 to 450 mW.

After establishing the parameters required to induce optimal orthogonal and reversible photoreactions, this knowledge was applied to the reversible photocycloadditions of quinolinones tethering macromolecules (**Figure** **6**A). Consequently, by following reported procedures,^[^ ^50^ ^]^ quinolinone scaffolds were monofunctionalized at the termini of a short molecular weight polyethylene glycol macromolecular chain (5 kD), denoted as PEG‐QM (Figure S13, Supporting Information), using straightforward urethane/carbamate post‐functionalization techniques. Note that PEG was chosen due to its common use as a matrix for demonstrating photoligation and its ease of modification.^[^ ^26^ ^]^ The functionalization could be confirmed by ^1^H‐NMR spectroscopic measurements (see Supporting Molecular Characterization) and further supported by GPC traces detected by UV–vis absorption at 340 nm (Figure S14, Supporting Information).

Based on the molecular studies, 340 nm was selected as a suitable irradiation wavelength to trigger the photoligation of the PEG‐QM whereas the photocycloreversion was triggered at 265 nm (Figure 6A). While the coupling of the macromolecular chains could not be confirmed by NMR analysis due to the low concentration of chromophores in the polymer matrices, GPC traces; however, disclosed a twofold increase in molecular weight (Figure S15, Supporting Information); thus, confirming the photo‐induced ligation. Note that the presence of a small quantity of this twofold species was noticeable in the reference samples PEG‐QM (Figure S14, Supporting Information), attributed to the component's instability in solution and partial cycloaddition initiation upon exposure to daylight. UV–vis absorption spectra also indicated a successful ligation by exhibiting a decrease in the characteristic band of QM at 328 nm (Figure 6A). The irradiation of PEG‐QM in acetonitrile (*c* = 5.28 × 10^−5^ mol L^−1^) at 340 nm demonstrated a maximum conversion of 92% after 20 min (Figure 6B,C). It is worth noting that the photoligation process of macromolecules required significantly more time (20 times longer) compared to the formation of molecular cyclobutanes. This decrease in reaction rates highlights the influence of the macromolecules on the photoreaction, presumably impeding the diffusion process. Nevertheless, despite the longer reaction time, the photoligation exhibited similar high conversions when compared to their molecular counterparts.

Following the successful demonstration of the ligation photoprocess on the macromolecular scale, the cyclability of the photoligation and cleavage was examined in a one‐pot reaction using the previously determined conditions. Absorption spectroscopy was utilized to monitor the reaction conversion for each photoprocess, employing irradiations at 340 nm for the cycloaddition and 265 nm for the cycloreversion. Gratifyingly, a similarly remarkable reversibility process, mirroring the behavior observed in the molecular structure, was obtained, with a reversion process occurring after 2 min of irradiation (Figure 6C). This cyclic photoprocess was supported by GPC traces, showing the recovery of the original molecular weight of PEG‐QM (Figure S15, Supporting Information). Consistent with the molecular studies, the residues of the solvent also appeared to impact the first cycle of the photoprocess, exhibiting a similar discrepancy of 20% after the initial cycle.

## 3 Conclusion

The pursuit of effective wavelength‐driven covalent gated chemistry, characterized by precise control and stable cyclability, remains a compelling focus for diverse applications. Yet, achieving this goal demands dedicated endeavors in the development of novel responsive motifs with such capabilities. In this study, we demonstrated that through a progressive study approach, we could leverage the photoperformance of quinolinones to achieve truly gated functions with degrees of reversibility never achieved before. Specifically, we demonstrated the need of conducting reactions under oxygen‐free conditions to prevent the occurrence of side reactions typically encountered in these systems, leading to improved yield (near 100%) and exclusive access to cyclable photoprocesses under these conditions. In addition, the individual study of each photoprocess (i.e., cycloaddition and cycloreversion) allowed the establishment of optimal parameters for maximizing the reaction in terms of speed, conversion, and control of the produced photoproducts. Moreover, it was demonstrated that screening the wavelength to study kinetics is a valuable tool not only for further optimizing photoperformance (speed/conversion) but also for achieving a high orthogonality of the process. The combination of the optimized photoprocesses revealed remarkable reversibility with negligible losses across eight cycles.

The comprehensive understanding acquired from studying individual photoprocessess also allowed us to concentrate on factors negatively influencing cyclability, in our case, particularly the ones occurring in the first irradiation cycle. The direct comparison with results obtained in the individual reversion study allowed us to decouple the observed decay from the photostationary state and bleaching event, usually attributed to that phenomenon, but instead, assigned to the catalytic amount of residues present in the solvent. Ultimately, the successful translation of these meticulously optimized photoprocesses to a macromolecular system not only demonstrated their outstanding performance but also emphasized the efficacy in initiating covalent gated photoligation within polymer chains. This proposed methodology and its practical application marks a significant step forward in leveraging the full potential of photochemically responsive motifs, such as quinolinones. Such approach ensures superior control and advancement in polymer functions, setting the groundwork for creating innovative and functional polymer materials for a range of applications.

## Conflict of Interest

The authors declare no conflict of interest.

## Supporting information

Supporting Information

## Data Availability

The data that support the findings of this study are available from the corresponding author upon reasonable request.
