# Supplementary material for: Harnessing the Photoperformance of N‐Methyl‐Quinolinone for Gated Photo‐Driven Cyclability and Reversible Photoligation
Source: Macromol Rapid Commun. 2024 Aug 3;45(22):2400474. doi: 10.1002/marc.202400474 (PMC11583344; doi:10.1002/marc.202400474)
Supplement: Supplementary file 1 — Supporting Information [file MARC-45-2400474-s001.docx]

DOI: 10.1002/((please add manuscript number))

**Article type: Research article**

**Harnessing the photoperformance of N-Methyl-Quinolinone for gated photo-driven cyclability and reversible photoligation**

Moritz Streicher^1†^, Claas-Hendrik Stamp^1†^, Marco Dante Kluth^2^, Alexander Ripp^1^, Céline Calvino^1,2^*

^1^M. Streicher, C.-H. Stamp, A. Ripp, C. Calvino

Cluster of Excellence livMatS, University of Freiburg (livMatS)

FIT-Freiburg Center for Interactive Materials and Bioinspired Technologies

Georges-Köhler-Allee 105, D-79110 Freiburg, Germany
E-mail: [C](mailto:christoph.weder@unifr.ch)eline.calvino@livmats.uni-freiburg.de

^2^M. D. Kluth, C. Calvino

University of Freiburg – Department of Microsystems Engineering (IMTEK)

Georges-Köhler-Allee 102, D-79110 Freiburg, Germany
E-mail: [Celine.calvino@livmats.uni-freiburg.de](mailto:Celine.calvino@livmats.uni-freiburg.de)

–––––––––

1. Set value for Heading 9

Table of Contents:

[1 Supporting Data S-2](#_Toc170369682)

[2 Supporting Experimental Section S-11](#_Toc170369683)

[3 Synthetic Procedures and Analytical Data S-16](#_Toc170369684)

[4 Supporting Molecular Characterization S-23](#_Toc170369685)

[5 References S-32](#_Toc170369686)

# 1 Supporting Data


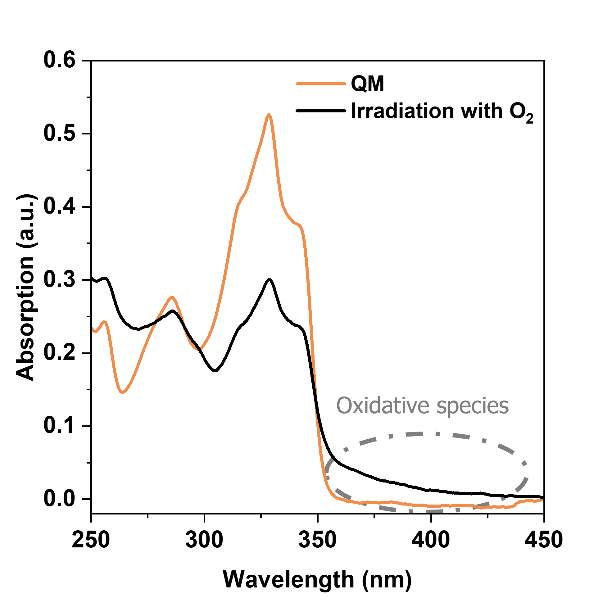


**Figure S1.** UV-vis absorption spectra of pure **QM** (orange) and 120 hours post-irradiation at 365 nm in the presence of oxygen (black). The characteristic absorption of the oxidation side reaction is highlighted within a shaded grey circle. Photoreactions were conducted in acetonitrile solutions at *c* = 5.28 mmol and irradiated with a LED photoreactor at a power of 450 mW.


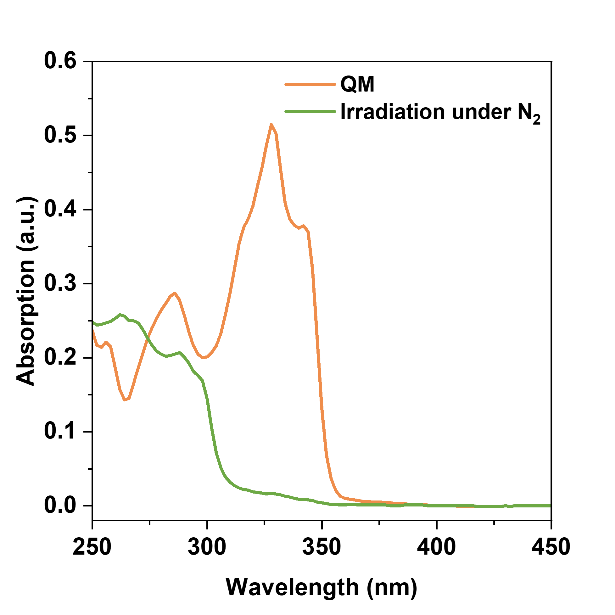


**Figure S2.** UV-vis absorption spectra of pure **QM** (orange) and 120 hours post-irradiation at 365 nm under nitrogen and oxygen free conditions (green). Photoreactions were conducted in acetonitrile solutions at *c* = 5.28 mmol and irradiated with a LED photoreactor at a power of 450 mW.


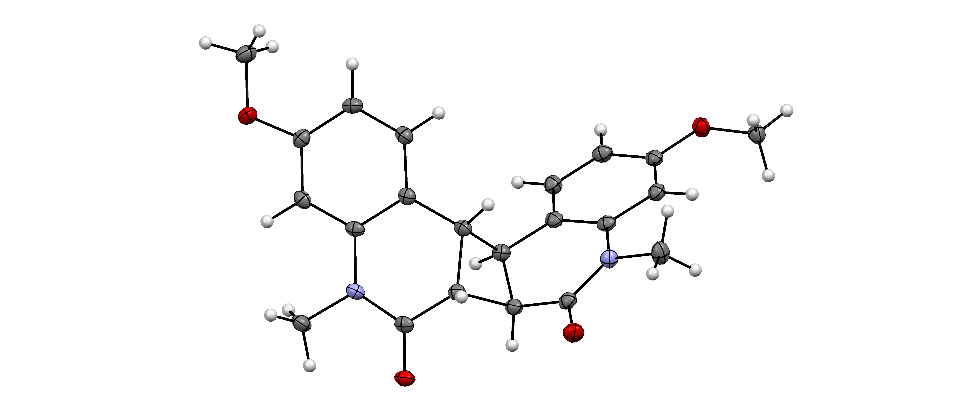


**Figure S3**. Crystal structure of anti Head-to-Head quinolinone dimer (**QD*_aHH_***) with thermal ellipsoids shown at a 50 % probability level. Grey, blue and red ellipsoids correspond to carbon, nitrogen and oxygen atoms, respectively. Crystals were obtained from acetonitrile at room temperature during irradiation of the solution with 340 nm LEDs. Data were collected from a shock-cooled single crystal at 100 K on a Bruker D8 VENTURE dual wavelength Mo/Cu three-circle diffractometer with a microfocus sealed X-ray tube using a mirror optics as monochromator and a Bruker PHOTON III detector. The diffractometer was equipped with an Oxford Cryostream 800 low temperature device and used Cu*K_α_* radiation (λ = 1.54178 Å). All data were integrated with SAINT and a multi-scan absorption correction using SADABS was applied.^[1,2]^ The structure was solved by direct methods using SHELXT and refined by full-matrix least-squares methods against *F*^2^ by SHELXL-2018/3.^[3]^ All non-hydrogen atoms were refined with anisotropic displacement parameters. All hydrogen atoms were refined isotropic on calculated positions using a riding model with their *U*_iso_ values constrained to 1.5 times the *U*_eq_ of their pivot atoms for terminal sp^3^ carbon atoms and 1.2 times for all other carbon atoms. Crystallographic data for the structures reported in this paper have been deposited with the Cambridge Crystallographic Data Centre.^[4]^ CCDC 2257859 contain the supplementary crystallographic data for this paper. These data can be obtained free of charge from The Cambridge Crystallographic Data Centre via www.ccdc.cam.ac.uk/structures. The report and the CIF file were generated using FinalCif.^[5]^ Further details can be found in **Table S1**.

**Table S1.** Crystal data and structure refinement.

| CCDC number | 2257859 |
| --- | --- |
| Empirical formula | C_22_H_22_N_2_O_4_ |
| Formula weight | 378.41 |
| Temperature [K] | 100(2) |
| Crystal system | triclinic |
| Space group (number) | $P\overline{1}$ (2) |
| *a* [Å] | 9.1672(9) |
| *b* [Å] | 9.1908(11) |
| *c* [Å] | 12.3835(14) |
| α [°] | 86.124(7) |
| β [°] | 71.484(4) |
| γ [°] | 62.566(4) |
| Volume [Å^3^] | 874.24(17) |
| *Z* | 2 |
| *ρ*_calc_ [gcm^−3^] | 1.438 |
| *μ* [mm^−1^] | 0.813 |
| *F*(000) | 400 |
| Crystal size [mm^3^] | 0.577×0.442×0.282 |
| Crystal colour | colourless |
| Crystal shape | block |
| Radiation | Cu*K_α_* (λ=1.54178 Å) |
| 2θ range [°] | 7.56 to 144.33 (0.81 Å) |
| Index ranges | −10 ≤ h ≤ 11 −10 ≤ k ≤ 11 −14 ≤ l ≤ 15 |
| Reflections collected | 58051 |
| Independent reflections | 3243 *R*_int_ = 0.0371 *R*_sigma_ = 0.0138 |
| Completeness to  θ = 67.679° | 97.4 % |
| Data / Restraints / Parameters | 3243/0/258 |
| Goodness-of-fit on *F*^2^ | 1.041 |
| Final *R* indexes  [*I*≥2σ(*I*)] | *R*_1_ = 0.0320 w*R*_2_ = 0.0816 |
| Final *R* indexes  [all data] | *R*_1_ = 0.0377 w*R*_2_ = 0.0855 |
| Largest peak/hole [eÅ^−3^] | 0.28/-0.21 |
| Extinction coefficient | 0.0089(7) |

**Figure S4.** UV-vis absorption spectra of pure **CM** (orange) and 120 hours post-irradiation at 365 nm under nitrogen and oxygen free conditions (green). Photoreactions were conducted in acetonitrile solutions at *c* = 5.28 mmol/L and irradiated with a LED photoreactor at a power of 450 mW.

**Figure S5.** Comparison of the photocycloaddition conversions of **QM** (squares), conducted under nitrogen and oxygen free conditions) and **CM** (triangles) conducted in presence of oxygen over time. Photoreactions were carried out in acetonitrile solutions at *c* = 5.28 mmol/L and irradiated with a LED photoreactor at a power of 450 mW.


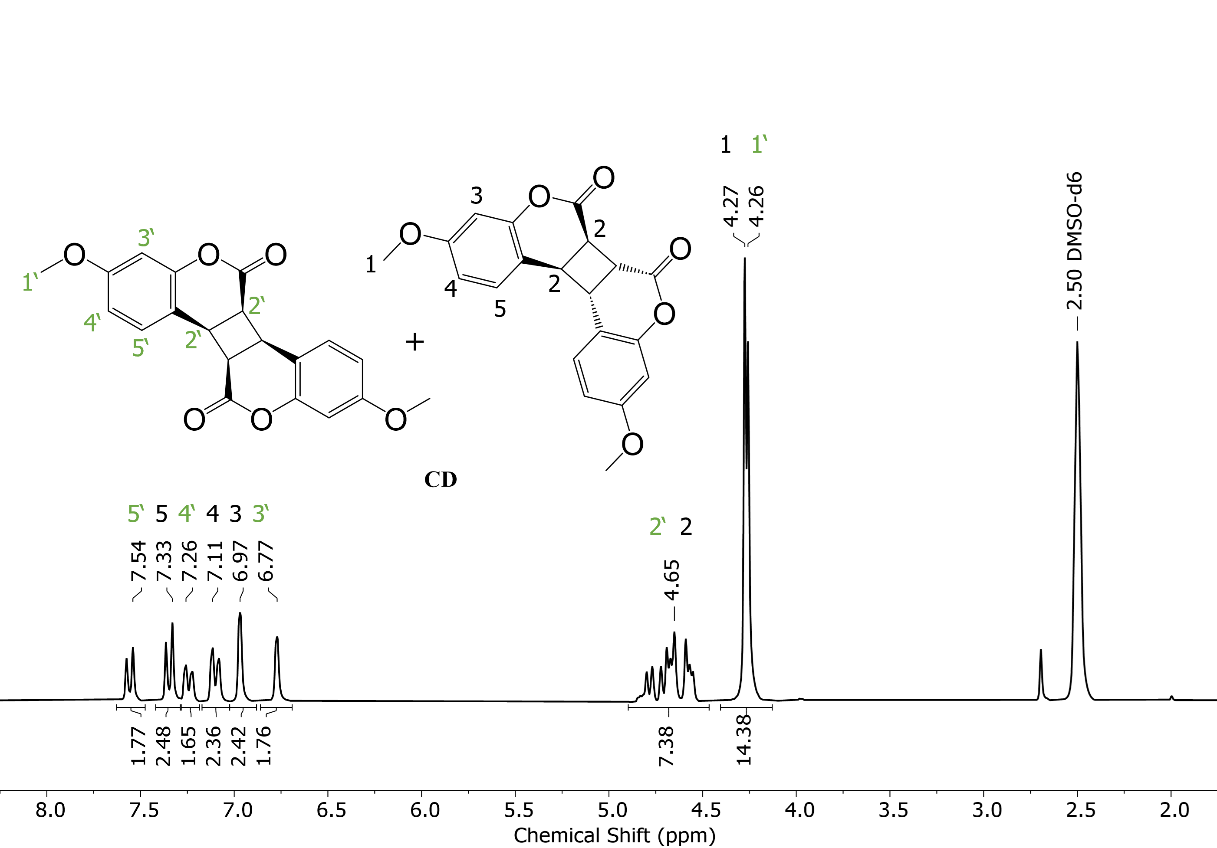


**Figure S6.** ^1^H-NMR spectra of **CM** after irradiation at 365 nm for 125 hours at *c* = 0.57 mol/L. The signals have been assigned to the formation of two isomers: **CD*_aHH_*** (60 %) and **CD*_sHT_*** (40 %).


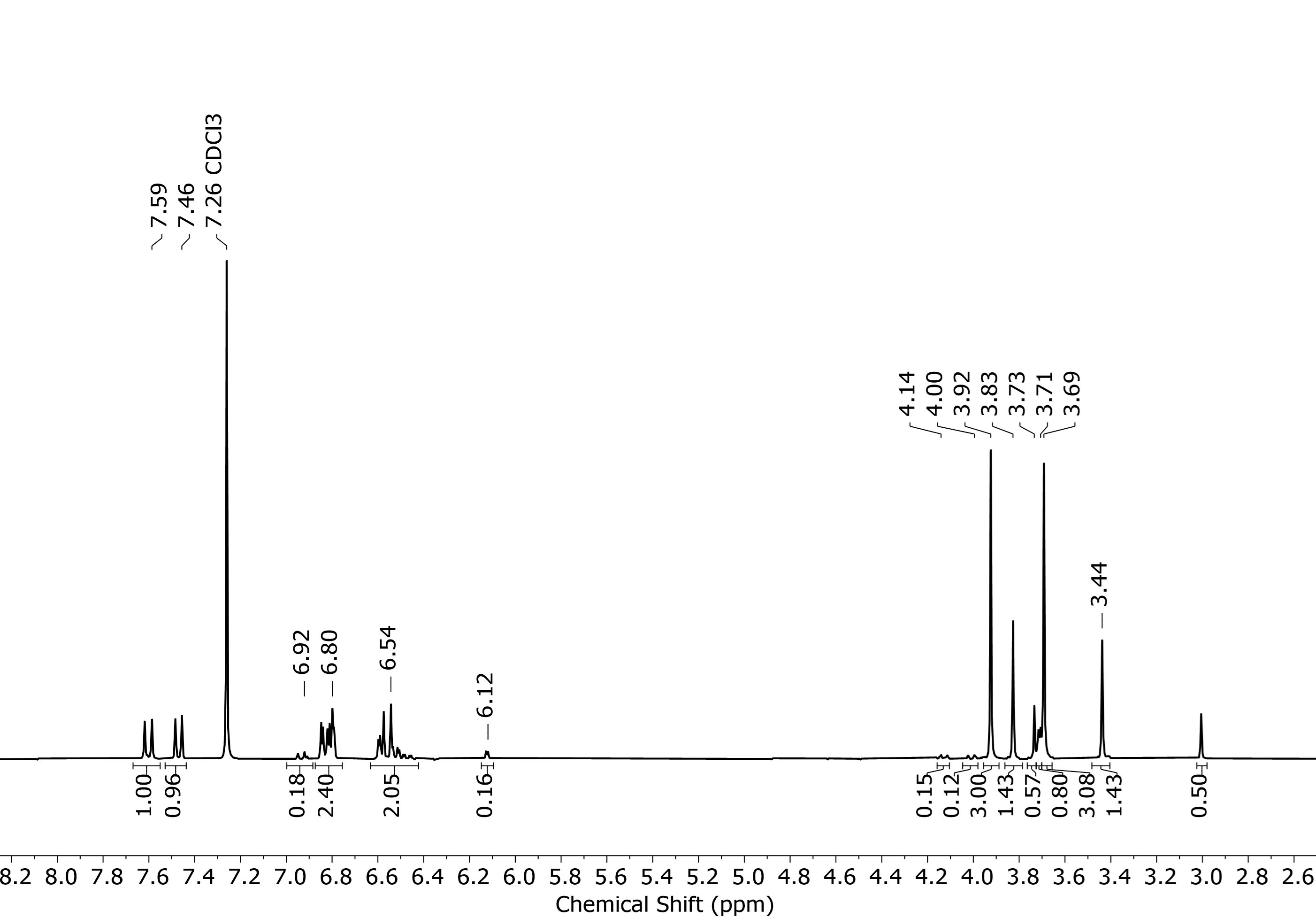


**Figure S7.** ^1^H-NMR spectra of **QM** after irradiation at 340 nm for 17.5 hours at *c* = 0.035 mol/L. The signals have been assigned to the formation of two isomers: **QD*_aHH_*** (74 %) and **QD*_sHT_*** (26 %).


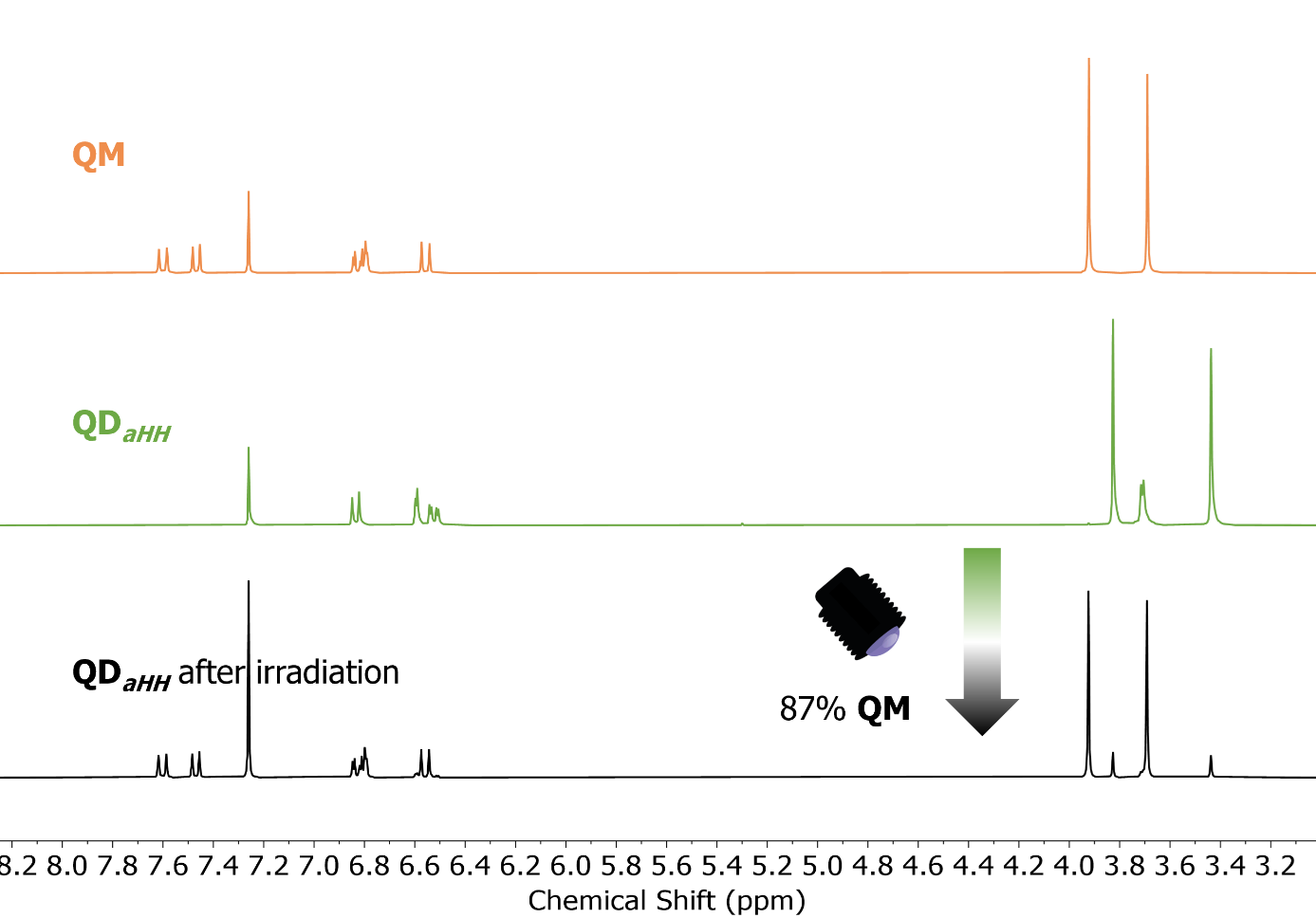


**Figure S8.** ^1^H-NMR analysis of a cycloreversion of **QD*_aHH_*** at 265 nm for 18 hours (black spectrum). **QD** was obtained with 87 % conversion upon irradiation. The ^1^H-NMR spectra of **QD*_aHH_*** (green) and **QM** (orange) are provided for reference. Photoreactions were conducted in acetonitrile solutions at *c* = 2.6 mmol/L and irradiated with a LED photoreactor at a power of 22 mW.


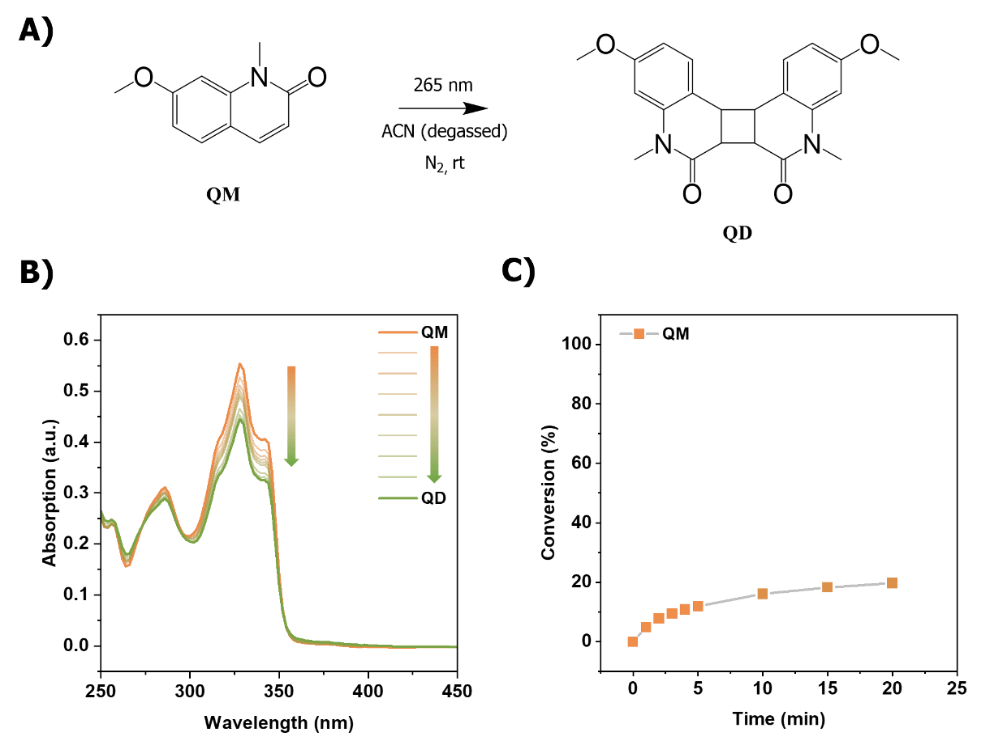


**Figure S9. A)** Schematic representation of the [2π+2π]-cycloaddition of **QM** upon irradiation at 265 nm. **B)** Corresponding UV-vis absorption spectra of the cycloaddition (orange to green), and **C)** the corresponding conversions. Photoreactions were conducted in acetonitrile solutions at *c* = 10^-5^ mol/L and irradiated with a LED photoreactor at a power of 22 mW.


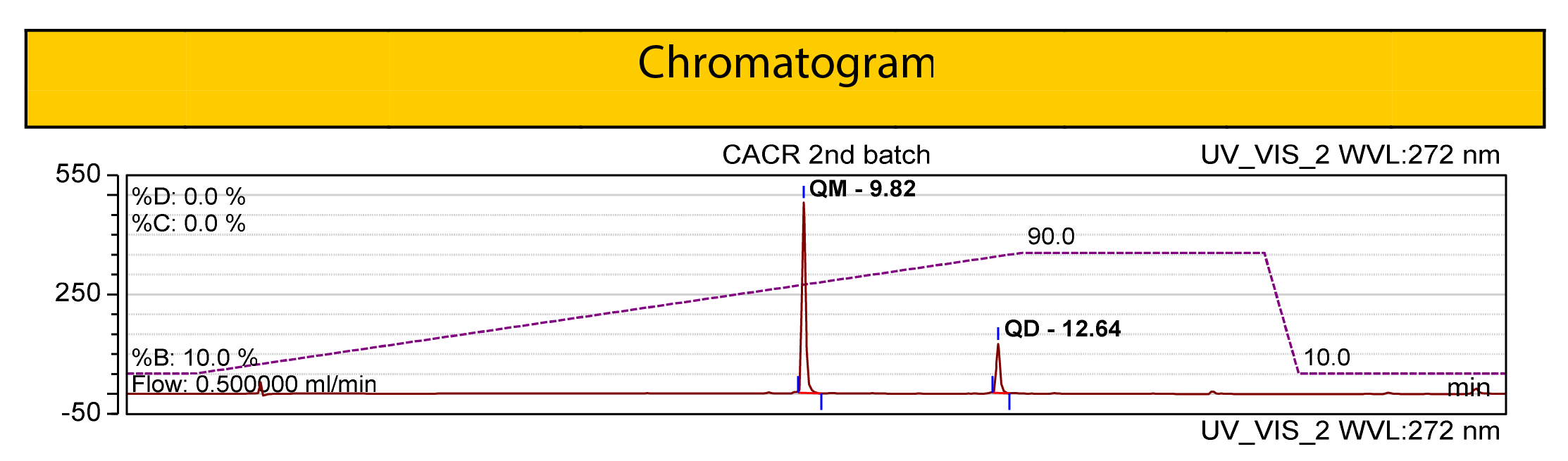


**Figure S10.** HPLC chromatogram of **QM** after a subsequent irradiation cycle at 340 nm and 265 nm.


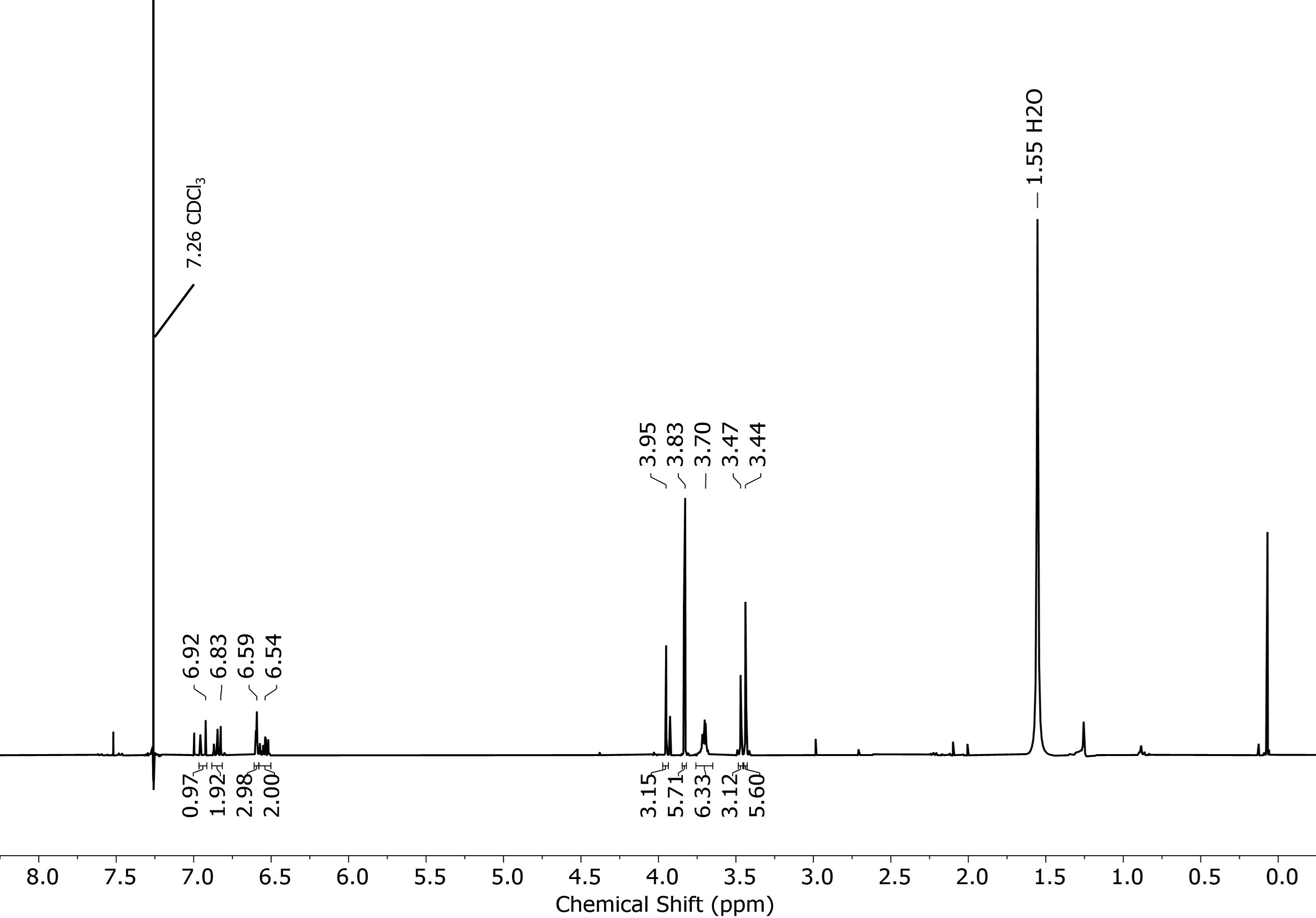


**Figure S11.** ^1^H-NMR spectra of isolated products after MPLC purification (Interchim, column: PF-30C18HP-F012, flow: 15 mL/min, gradient: water (A) against MeCN (B), 0CV: 30 % B, 1CV: 30 % B, 10CV: 85 % B, 12CV: 85 % B) and lyophilization.


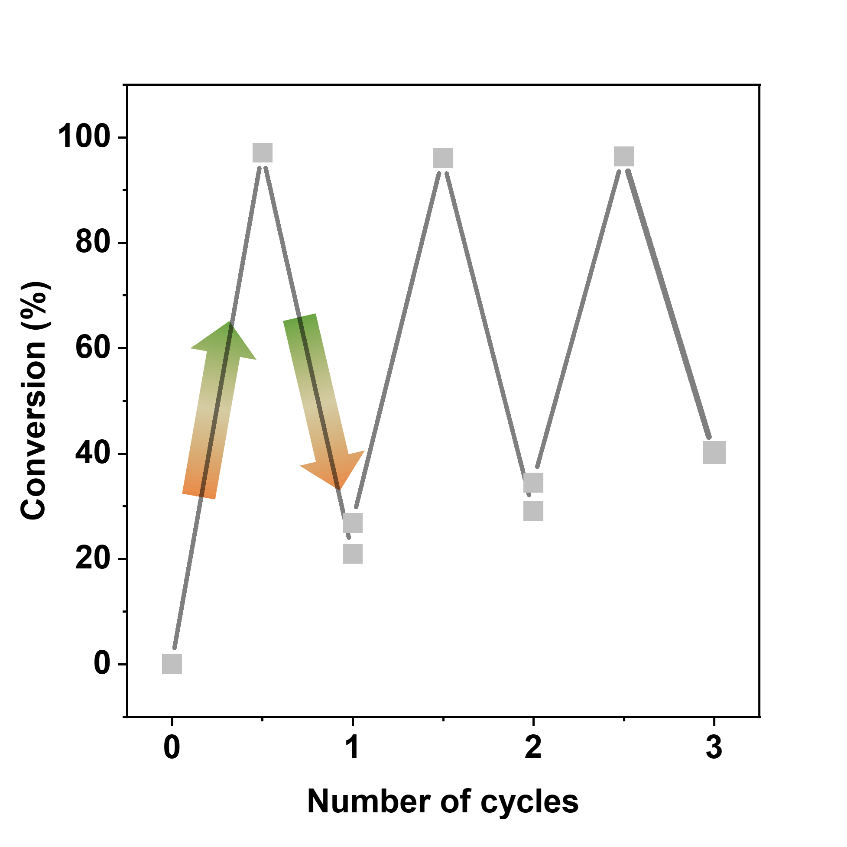


**Figure S12.** Conversion of **QM** after subsequent cycles of cycloaddition (CA) and reversion (CR). After each CA/CR cycles the solvent in the cuvette was evaporated and replaced by fresh acetonitrile, reintroducing fresh impurities in the reaction and further decreasing its conversion. This procedure was repeated two times to generate the second third cycle. The photoreactions were conducted at irradiation wavelengths of 340 nm and 265 nm (*c* = 10^-5^ mol/L), respectively, and irradiated with a LED photoreactor at a power ranging between 22 and 450 mW.

**Figure S13.** Synthetic pathway of PEG mono end-functionalized with quinolinone (**PEG-QM**).

**
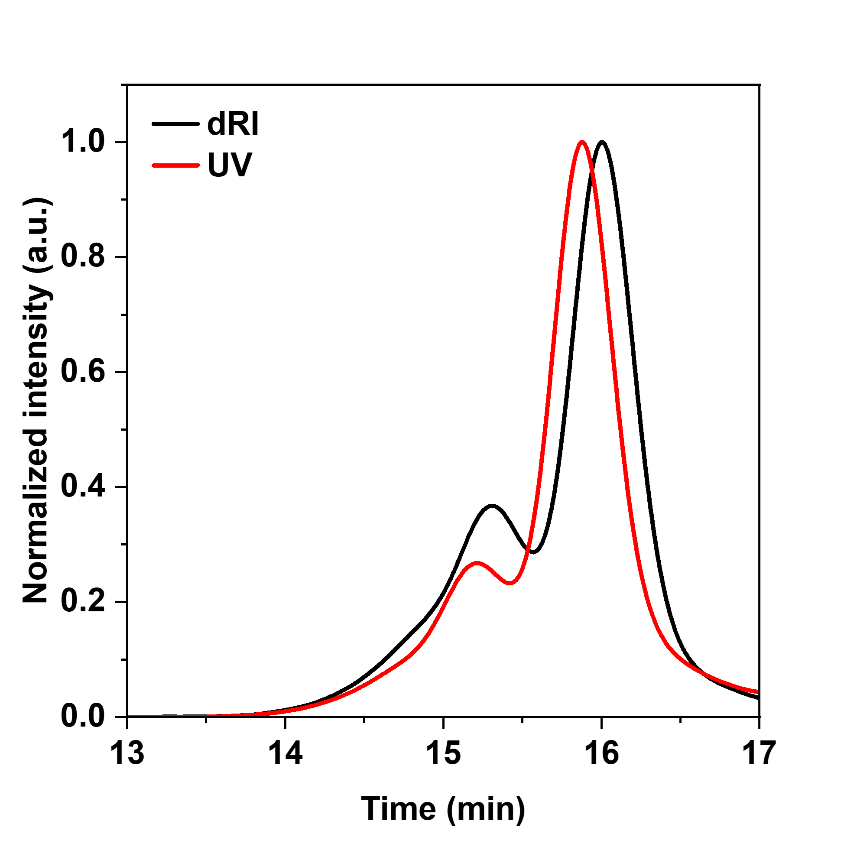
**

**Figure S14.** Size exclusion chromatography (SEC) traces of **PEG-QM** with the refractive index difference (dRI, black trace) and the UV detector signal (red trace). The number-average molecular weight (M_n_) was determined to be 12.915 g/mol at a retention time of 15.3 min and 5.280 g/mol at a retention time of 16 min, calibrated against a poly(ethylene glycol) standard.


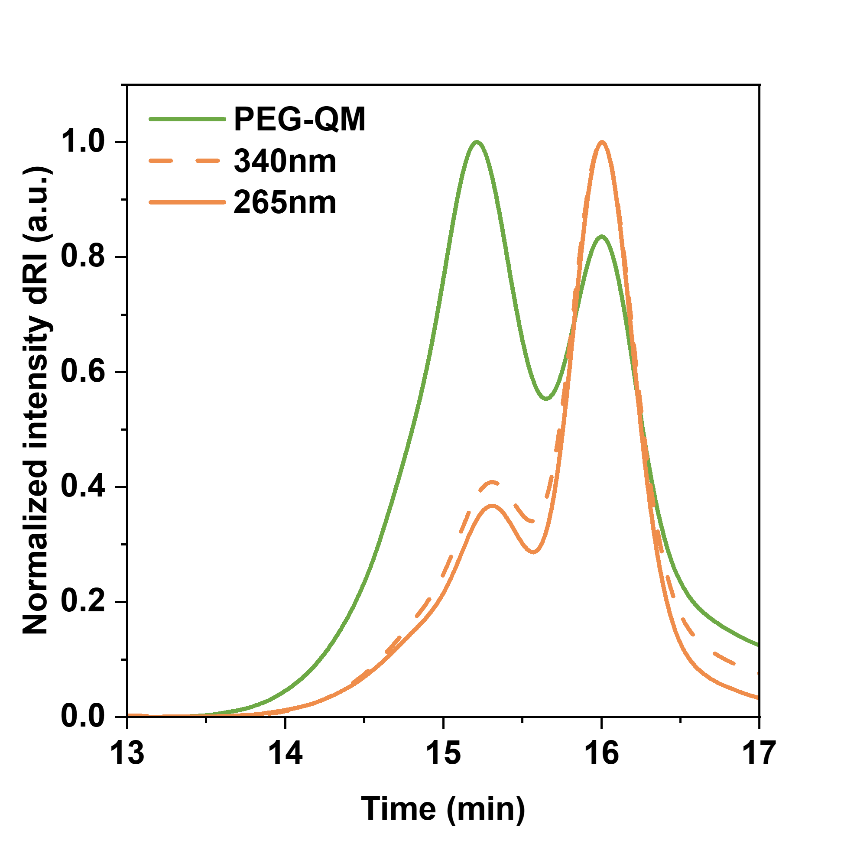


**Figure S15.** Size exclusion chromatography (SEC) traces of **PEG-QM** with the refractive index difference (dRI, black trace) of neat **PEG-QM** (orange), **PEG-QM** after 20 min irradiation at 340 nm (green) and after a subsequent 3 min irradiation at 265 nm (dashed orange). The number-average molecular weight (M_n_) was determined to be 12.915 g/mol at a retention time of 15.3 min and 5.280 g/mol at a retention time of 16 min, calibrated against a poly(ethylene glycol) standard. Photoreactions were conducted in acetonitrile solutions at *c* = 10^-5^ mol/L and irradiated with a LED photoreactor at powers ranging from 22 to 450 mW.

# 2 Supporting Experimental Section

## 2.1 Materials and Instrumentation

**Materials**

All reagents and solvents were used without further purification, if not specified otherwise. N,N-dimethylformamide (>99.5 %), ethyl acetate (>99 %), dichloromethane (>99.5 %), cyclohexane (>99.5 %), acetone (puriss), acetonitrile (>99.5 %), diethyl ether (100 %) and toluene (>99.5 %) as well as acetonitrile-*d*_3_ (99.8 %), dimethylsulfoxide-*d*_6_ (99.8 %) and chloroform-*d* (99.8 %) were purchased from Carl Roth. Anhydrous potassium carbonate (99 %), iodomethane (99 %), 11-bromoundecanol (97 %), methoxypolyethylene glycol amine (M*_w_* = 5’000 g/mol), 1,6-diisocyanatetohexane (99 %) were purchased from Thermofisher Scientific. Pyridine hydrochloride (98 %), 7-hydroxycoumarin (Umbelliferone, 99 %), sodium hydride (60 % dispersion in paraffin wax) and 1,3,5-trioxane (for synthesis) were purchased from Sigma Aldrich. Dibutyltin-dilaureate (95 %) was purchased from Alfa Aesar. Dichloromethane and N,N-dimethylformamide were distilled and stored in molecular sieves (size = 4 Å).

**Nuclear magnetic resonance spectroscopy**

NMR Spectroscopy was carried out at 297 K on spectrometers from Bruker: Avance II 400 MHz, Avance II 300 MHz. Spectra were calibrated to the residual solvent peaks of acetonitrile-*d_3_*, dimethylsulfoxide-*d_6_* and chloroform-*d* at 1.94 ppm, 2.50 ppm and 7.26 ppm, respectively.^[6]^ Data were treated with MestReNova (14.1) software suite and all chemical shifts (δ) are reported in parts per million (ppm) with coupling constant in Hz (multiplicity: s = singlet, d = doublet, dd = doublet of doublet, t = triplet, dt = doublet of triplet, ddd = doublet of doublet of doublet, sep = septet, m = multiplet, br = broad signal).

**High-performance liquid chromatography**

A Thermo Scientific Dionex UltiMate 3000 setup for HPLC analysis equipped with Phenomenex Luna Phenyl-Hexyl (150 x 4.6 mm, dp: 3 µm) was employed to measure the chemical transformations of quinolinone into its respective dimers. For analysis, each irradiated product was dissolved in a mixture of acetonitrile and water (1:1, v:v), supplemented with 0.05 % trifluoroacetic acid, to obtain a concentration of approx. 1 mg/mL. The eluting solvent system consists of acetonitrile (B) against water (A) as the following gradient: 0 min: 30 % B, 1 min: 30 % B, 13 min: 85 % B, 16.5 min 85 % B, 17 min 30 % B. All solvents contained 0.1 % trifluoroacetic acid and the flow rate was set at 0.75 mL/min. Chromatograms were monitored at 272 nm and the relative areas under the peaks were used to identify the percentage of products’ conversion.

**Ultraviolet/visible light absorption spectroscopy**

UV-vis spectra were recorded on a Shimadzu UV-1800 spectrophotometer and data were acquired directly from the instrument in a CSV format. Quartz cuvettes (1 cm) from Thor Labs were used to measure the absorption from 190–500 nm, with 2 nm steps at a slow scan rate.

**Size exclusion chromatography (SEC).**

SEC experiments were performed on an Agilent 1260 Infinity II system equipped with one Agilent PolarGel M guard column (particle size = 8 μm) and two Agilent PolarGel M columns (ID = 7.5 mm, L = 300 mm, particle size = 8 μm). Signals were recorded by an interferometric refractometer (Agilent 1260 series). Measurements were conducted using 0.05 mol/L LiBr in DMF as the eluent, at a temperature of 50 °C, and with a flow rate of 1.0 mL/min. Molecular weights were determined based on narrow molecular weight poly(ethylene oxide) calibration standards.

**Photoreactor**

Irradiations were carried out with Atlas Photonics Lumos 43 photoreactors equipped with LEDs of various wavelengths (ca. 5 nm half width). The power associated with the utilized LED is indicated in the following table.

**Table S2**: LED power.

| Wavelength (nm) | 265 | 285 | 310 | 325 | 340 | 365 | 385 | 405 |
| --- | --- | --- | --- | --- | --- | --- | --- | --- |
| Power (mW) | 22 | 60 | 80 | 40 | 33 | 450 | 480 | 1300 |

This instrument is equipped with a power supply, a reactor and sample holders for standard NMR tube, test tube, UV-cuvette (1 cm).

## Methods and Procedures

### 2.1.1 General procedure for photoreactions

A stock solution of the adduct in acetonitrile (ACN) (1 mg/mL) was introduced into a glass vial and sealed with a rubber septum. 35 μL of this solution was then carefully transferred into a quartz cuvette and subsequently placed under high vacuum to evaporate the solvent. Meanwhile, additional ACN was degassed by purging nitrogen gas through the solvent for a duration of 20 minutes minimum. 3 mL of the degassed ACN was combined with the dried sample, under nitrogen atmosphere, to obtain an oxygen free and diluted solution of a concentration of ca. 10^‑5^ mol/L. The cuvette was sealed using a Teflon stopper under nitrogen stream, and parafilm was employed to seal the cuvette.

### 2.1.2 Cycloaddition

Cycloaddition reactions were carried out within the concentration range of 4-6·10^-5^ mol/L. The cuvettes were placed in the photoreactor using a sample holder. Irradiations were conducted at either 340 nm or 365 nm, and UV-vis spectra were recorded at intervals of 10 seconds to 20 minutes until a plateau was achieved, typically taking 1 to 80 minutes. It is worth noting that due to the experimental setup, real-time measurements could not be performed, and the irradiation kinetics needed to be momentarily halted for measurement purposes. Furthermore, to mitigate the influence of ongoing irradiation during UV-vis measurement, absorptions were recorded at the quinolinone's maximum absorption wavelength, specifically *λ* = 328 nm. Full spectral measurements were exclusively conducted for the reference spectra (sample at *t* = 0 min before irradiation), allowing for the subsequent conversion calculations. The conversion *c_t_* at time *t* was calculated by the following equation:

$$c_{t}=\left( 1-\frac{A_{t}}{A_{0}} \right)\cdot100 \%$$

where $A_{t}$ and $A_{0}$ are the absorptions at time $t$ and $t=0 \min$, respectively. The absorptions of quinolinone were measured at 328 nm, while the coumarin absorptions were measured at 316 nm.

### 2.1.3 Reversion

Reversion reactions were carried out within the concentration range of 2-3·10^-5^ mol/L. The cuvettes were placed in the photoreactor using a sample holder. Irradiations were conducted at either 265 nm, 285 nm or 310 nm, and UV-vis spectra were recorded at intervals of 5 to 10 seconds until a plateau was achieved, typically taking 3 to 6 minutes. Absorption measurements were conducted following the same procedure described for the cycloaddition (section 0). The conversion *c_t_* at time *t* was calculated by the following equations:

$$c_{t}=\left( 1-\frac{A_{t}}{A_{\max}} \right)\cdot100 \%$$

where the absorption for full conversion $A_{\max}$was determined by the following equation:

$$A_{max, 328}= \varepsilon_{M, 328}\cdot\left( 2\cdot\left( \frac{A_{0, 288}^{*}}{\varepsilon_{D, 288}} \right) \right)$$

Where $A_{0}^{*}$ is the initial absorption at 288 nm before irradiation and $\varepsilon_{M, 328}$ and $\varepsilon_{D, 288}$ are the extinction coefficients of the monomers at 328 nm and of the dimer at 288 nm, respectively.

### 2.1.4 Photocycling

Following the aforementioned sampling procedure, sequential execution of cycloaddition and cycloreversion processes was performed at 340 and 265 nm, respectively. Each irradiation step was conducted until reaching the absorption plateau, which typically occurred after approximately 10 minutes for cycloaddition and 2 minutes for cycloreversion. This cycle was iterated between 5 to 8 times. To streamline the process, some experiments only documented a single data point during these instances of maximum conversion.

### 2.1.5 Reversible photopolymerizations

Photopolymerizations were carried out as described in section 2.2.1-4 with similar molar concentrations (10^-5^ mol/L) compared to molecular studies.

### 2.1.6 Wavelength dependent photon efficiency analysis (WPEA)

Samples for WPEA experiments were prepared according to the abovementioned procedure under nitrogen and oxygen free conditions. The irradiations were carried out based on the conditions outlined in **table S3.** The photon flux $q_{p,\lambda}$ corresponding to each wavelength $\lambda$ was estimated by utilizing the output power $p_{\lambda}$ of the associated LEDs, with the assumptions of constant output power from the light sources during measurements, and the consideration that all emitted photons enter the reaction mixture. Under these assumptions, the photon flux $q_{p,\lambda}$ is expressed as:

$$q_{p,\lambda}=\frac{p_{\lambda}\cdot\lambda}{h\cdot c\cdot N_{A}}$$

where $h$ is the length of the light path through the cuvette, $c$ the concentration of the chromophore solution and $N_{a}$ the Avogadro constant. The reaction time $t_{\lambda}$ was then adjusted to reach a fixed number of photons $N_{p}$ by:

$$t_{\lambda}=\frac{N_{p}}{q_{p,\lambda}}$$

**Table S3**: Number of photons targeted during WPEA.

| Number of photons | **QM** | **CM** |
| --- | --- | --- |
| Photocycloaddition | 3.22·10^20^ | - |
| Photocycloreversion | 3.66·10^21^ | 8.80·10^20^ |

# 3 Synthetic Procedures and Analytical Data

### Synthesis of 7-methoxycoumarin (CM).

7-methoxycoumarin (**CM**) was synthesized according to reported procedures.^[7]^ Umbelliferone (1.95 g, 12.00 mmol, 1.0 eq) was suspended in N,N-dimethylformamide (16 mL) in a dried Schlenk flask and under nitrogen atmosphere. The suspension was cooled to 0 °C and sodium hydride (60 % suspension in paraffin wax) (0.49 g, 12.20 mmol, 1.1 eq.) was slowly added in the solution mixture. Iodomethane (0.75 mL, 1.71 g, 12.20 mmol, 1.1 eq.) was then added dropwise. The reaction mixture was allowed to warm up to room temperature and subsequently stirred for 2 h. Upon completion of the reaction, the solvent was removed under reduced pressure and the residue was dissolved in ethyl acetate (100 mL). The undissolved salts were removed by filtration, and the organic phase was washed with water (3 x 100 mL), brine (1 x 100 mL), and dried over magnesium sulfate. The dried organic phase was filtered, evaporated, and dried under reduced pressure. The crude product was purified by flash column chromatography using cyclohexane / ethyl acetate (7:3) as eluent to afford **CM** (1.29 g, 7.30 mmol, 61 %) as pale yellow solid. ^1^H-NMR (300 MHz, DMSO-*d*_6_) δ (ppm): 7.99-7.95 (d, 1H, vinyl ‑C*H* ), 7.62-7.59 (d, 1H, Ar*H*), 6.97-6.91 (m, 2H, Ar*H*), 6.29-6.26 (d, 1H, vinyl ‑C*H*), 3.84 (s, 3H, OC*H_3_*). ^13^C-NMR (75 MHz, DMSO-*d*_6_) δ (ppm): 162.45, 160.28, 155.42, 144.30, 129.44, 112.45, 112.35, 112.32, 100.67, 55.90. Melting point (DSC, 10 K/min.): 118 °C.

### Synthesis of 7-methoxycoumarin dimer (CD).

In a 100 mL round bottom flask, a 100 mg/mL solution of **CM** (5.00 g, 28.40 mmol) in acetonitrile (50 mL) including 1,3,5-trioxane (0.49 g, 5.40 mmol, 0.2 eq) as internal standard was degassed by N_2_ bubbling for 15 min. The photocycloaddition reaction was carried out under light irradiation at 365 nm using a Mightex High Power LED (450 mW) located at a 10 mm distance from the reaction vessel. The reaction was monitored by ^1^H-NMR, using 0.1 mL of reaction mixture diluted in 0.4 mL of deuterated acetonitrile. After 5 days, the solvent was evaporated under reduced pressure and the resulting product mixture was purified by flash column chromatography, using ethyl acetate / cyclohexane (1:1) as eluent, to afford a mixture of **CD*_sHT_*** and **CD*_sHH_*** isomers. (**CD*_sHT_***:**CD*_sHH_*** 1:1.4, 2.1 g, 6.00 mmol, 42 %) as a white solid. **CD*_sHT_* :** ^1^H NMR (300 MHz, DMSO-*d*_6_) δ (ppm): 6.95-6.92 (d, 2H, Ar*H* ), 6.72-6.68 (dd, 2H, Ar*H* ), 6.30-6.29 (d, 2H, Ar*H* ), 4.23-4.18 (m, 4H, cyclobutane -C*H*-), 3.67 (s, 6H, -OC*H_3_*) ^13^C NMR (75 MHz, DMSO-*d*_6_) δ in ppm:164.14, 159.64, 151.43, 129.72, 110.96, 110.09, 101.60, 55.40 (2C), 39.94, 38.78. **CD*_sHH_*:** ^1^H NMR (300 MHz, DMSO-*d*_6_) δ (ppm): 6.81-6.79 (d, 2H, Ar*H*), 6.58-6.55 (dd, 2H, Ar*H*), 6.46-6.45 (d, 2H, Ar*H*), 4.10 (s, 4H, cyclobutane -C*H*-), 3.68 (s, 6H, -OC*H_3_*). ^13^C NMR (75 MHz, DMSO-*d*_6_) δ (ppm): 164.95, 159.64, 152.54, 130.12, 110.77, 109.95, 101.48, 55.39 (2C), 36.35. **CD*_sHT_*, CD*_sHH_* mixture:** Melting point (DSC, 10 K/min.): 168 °C.

### Synthesis of 7-methoxy-1-methylquinolin-2-one (QM).

7-methoxy-1-methylquinolin-2-one (**QM**) was synthesized according to previously reported procedures.^[7]^ 1-Hydoroxyquinolin-2(1H)-one (3.00 g, 18.60 mmol, 1.0 eq) was suspended in dimethylformamide (30 mL) in a flame-dried 100 mL Schlenk flask under nitrogen atmosphere. The suspension was cooled to 0 °C, and sodium hydride (60 % suspension in paraffin wax) (1.64 g, 40.96 mmol, 2.2 eq.) was slowly added. The suspension was stirred for 15 min at 0 °C, followed by a dropwise addition of iodomethane (2.55 mL, 5.81 g, 40.96 mmol, 2.2 eq.). The reaction mixture was allowed to warm to room temperature and stirred for 2 hours. Upon completion of the reaction (TLC using EtOAc:CyH (6:4) as eluent), the solvent was removed under reduced pressure. The residue was dissolved in ethyl acetate (150 mL) and washed with water (150 mL) and subsequently with brine (150 mL). The organic layer was dried over magnesium sulfate, filtered, evaporated and fully dried under reduced pressure. The crude product was purified by flash column chromatography, using EtOAc:CyH 6:4 as eluent, to afford **QM** (2.78 g, 14.70 mmol, 79 %) as a white solid. ^1^H-NMR (300 MHz, CDCl_3_) δ (ppm): 7.61-7.58 (d, 1H, vinyl -C*H*), 7.48-7.45 (d, 1H, Ar*H*), 6.84–6.78 (m, 2H, Ar*H*), 6.57-6.54 (d, 1H, vinyl -C*H*), 3.92 (s, 3H, -OC*H_3_*), 3.68 (s, 3H, -NC*H_3_*). ^13^C-NMR (75.4 MHz, CDCl_3_) δ (ppm): 162.89, 161.93, 141.87, 138.85, 130.23, 118.67, 115.05, 109.72, 98.80, 55.73, 29.59. Melting point (DSC, 10 K/min.): 101 °C. HRMS, ESI, (m/z) [M—Na]^+^ calc. for C_11_H_11_NO_2_: 190.0868); found: 190.0864.

### Synthesis of 7-methoxy-1-methylquinolin-2-one anti-head-to-head dimer (QD*_aHH_*)

**QM**

(33 mg, 0.17 mmol) was dissolved in degassed acetonitrile (0.5 mL) inside an NMR tube under a nitrogen atmosphere. The solution was then irradiated using a 340 nm LED for 18 hours. The precipitate formed during the reaction was filtrated out the solvent to afford **QD*_aHH_*** (26 mg, 0.14 mmol, 79 %) as white crystals.

^1^H-NMR (300 MHz, CDCl_3_) δ  (ppm): 6.84-6.81 (d, 2H, Ar*H*), 6.59-6.58 (d, 2H, Ar*H*), 6.53-6.50 (dd, 2H, Ar*H*), 3.82 (s, 6H, -OC*H*_3_), 3.70-3.69 (m, 4H, cyclobutane-C*H*), 3.42 (s, 6H, -NC*H*_3_). ^13^C NMR (75 MHz, CDCl_3_) δ  (ppm): 169.45, 159.88, 140.87, 128.55, 116.12, 106.87, 102.88, 55.57, 43.92, 43.54, 29.83. HRMS, ESI, (m/z) [M—Na]^+^ calc. for C_22_H_22_N_2_O_4_: 401.1472; found: 401.1474.

### Synthesis of 7-hydroxy-1-methylquinolin-2-one (1)

**QM** (1.3 g, 7.14 mmol) and pyridinium hydrochloride (4.1 g, 35.72 mmol, 5.0 eq) were combined and well mixed in a 35 mL microwave vial. The reaction was performed under microwave irradiation (193 °C, 50 W) under high stirring for 1.5 h. The resulting brown solid was then poured into iced water (100 mL) under stirring. The suspension was isolated by filtration, and washed with additional water (50 mL). The remaining solid was dried under high vacuum to afford **1** (863 mg, 4.93 mmol, 69 %) as a white powder. ^1^H NMR (300 MHz, DMSO-*d*_6_) δ  (ppm): 10.25 (s, 1H, -O*H*), 7.76-7.73 (d, 1H, vinyl -C*H*-), 7.53-7.50 (d, 1H, Ar*H*), 6.80–6.70 (m, 2H, Ar*H*), 6.36-6.33 (d, 1H, vinyl -C*H*-), 3.52 (s, 3H, -NC*H_3_*). ^13^C NMR (75.4 MHz, DMSO-*d*_6_) δ  (ppm): 161.49, 160.16, 141.69, 139.11, 130.31, 116.62, 113.17, 111.08, 100.00, 28.86. Melting point (DSC, 10 K/min.): 224 °C.

### Synthesis of 7-(2-hydroxyethoxy)-1-methylquinolin-2-one (2)

Compound (**1**) (1.5 g, 8.50 mmol, 1 eq) and K_2_CO_3_ (2.3 g, 17.00 mmol, 2.0 eq) were dissolved in dry DMF (35 mL) in a flame dried Schlenk flask (100 mL), under nitrogen atmosphere. The reaction mixture was heated to 80°C and stirred for 15 minutes before adding dropwise 2-Bromoethanol (2.12 g, 1.20 mL, 17.00 mmol, 2.0 eq), and then further stirred at 80 °C for 22 h. Upon completion of the reaction (TLC using EtOAc as eluent), the solvent was fully evaporated under reduced pressure. The residue was dissolved in EtOAc and purified by silica gel flash column chromatography using EtOAc as eluent to afford **2** (1.60 g, 7.30 mmol, 86 %) as a pale orange solid. ^1^H NMR (300 MHz, DMSO-*d_6_*) δ (ppm): 7.82-7.79 (d, 1H, vinyl-C*H*), 7.64-7.61 (d, 1H, Ar*H*), 6.94-6.88 (m, 2H, Ar*H*), 6.43-6.40 (d, 1H, vinyl-C*H*), 4.95-4.91 (t, 1H, -O*H*), 4.16-4.13 (t, 2H, O-CH_2_C*H_2_*OH), 3.79-3.74 (m, 2H, O-*CH_2_*CH_2_OH), 3.59 (s, 3H, -NC*H_3_*). ^13^C NMR (75.4 MHz, DMSO-*d*_6_) δ  (ppm): 161.43, 160.95, 141.45, 138.93, 130.14, 117.59, 114.12, 110.17, 99.31, 70.00, 59.50, 29.01. HRMS, ESI, (m/z) [M—Na]^+^ calc. for C_12_H_13_NO_3_Na: 242.08; found: 242.0788.

### Synthesis of Q-HMDI (3)

Compound **2** (0.1 g, 0.46 mmol, 1 eq) was placed in a flame dried 25 mL round-bottom flask under nitrogen atmosphere and fully dissolved in 5 mL of dry dichloromethane. Heating was applied to properly dissolve the component. Upon full dissolution, 1,6-diisocyanatohexane (0.58 mL, 3.65 mmol, 8 eq) was quickly injected, immediately followed by one drop of dibutyltin-dilaureate (DBTDL). The reaction mixture was stirred under nitrogen atmosphere for 1 h at 50°C. The reaction was then allowed to cool to room temperature and the solvent was reduced to half its volume under reduced pressure. The resulting mixture was then precipitated in cold toluene to afford **Q-HMDI** (**3**) (60 mg, 0.15 mmol, 66 %) as a white solid.

Despite the observation of a few impurities in the spectra (excess of HMDI), this compound was used without further purification in the subsequent step. ^1^H NMR (400 MHz, DMSO-*d_6_*) δ (ppm): 7.83-7.80 (dd, 1H, vinyl-C*H*), 7.64-7.62 (dd, 1H, Ar*H*), 7.26 (t, 1H, carbamate-*H*), 6.95 (t, 1H, Ar*H*), 6.91-6.88 (dt, 1H, Ar*H*), 6.44-6.41 (dd, 1H, vinyl-C*H*), 4.31 (s, 4H, O-C*H_2_*-C*H_2_*-O), 3.59 (s, 3H, NC*H_3_*), 3.34-3.31(m, 2H, C*H_2_*-NCO), 3.00 – 2.91 (m, 2H, C*H_2_*-carbamate), 1.60-1.52-1.22 (m, 8H, C*H_2_*-C*H_2_*-C*H_2_*-C*H_2_* from hexyl-carbamate). ^13^C NMR (75.4 MHz, DMSO-*d*_6_) δ  (ppm): δ 161.39, 160.43, 158.03, 155.98, 141.45, 138.90, 130.21, 117.78, 114.30, 110.07, 99.30, 66.78, 62.17, 42.46, 30.44, 29.99, 29.34, 29.19, 29.05, 26.06, 25.98, 25.63, 25.53.

### Synthesis of PEG-QM

Methoxypolyethylene glycol amine, (CH_3_-O-PEG-NH_2_) (100 mg, 0.02 mmol, 1 eq) was placed in a 20 mL round bottom flask under vacuum and slow stirring for 4 h. The polymer was then dissolved in dry dichloromethane and combined with a dry solution of **3** (0.9 mg, 0.024 mmol, 1.2 eq) in dichloromethane (3 mL). The reaction mixture was stirred overnight under nitrogen atmosphere. The mixture was filtered and precipitated three times in hexane to yield **PEG-QM**. (30 mg, 0.005 mmol, 27 %). ^1^H NMR (400 MHz, DMSO-*d_6_*) δ 7.82-7.80 (d, 1H, vinyl-C*H*), 7.64-7.62 (d, 1H, Ar*H*), 6.96-9.95 (t, 1H, Ar*H*), 6.91-6.88 ( 1H, Ar*H*), 6.44-6.41 (d, 1H, vinyl-C*H*), 4.31 (s, 4H, -OC*H*_2-_C*H*_2_-O-), 3.71-3.67 (t, 2H, C*H_2_*-OCH_3_), 3.59 (s, 3H, N-C*H*_3_), 3.14 -3.14-3.11 (t,, 2H, NH-C*H_2_*), 2.97 -2.93 (m, 4H, NH-CO-NH-C*H_2_),* 1.38-1.15 (m, 10H, HMDI-spacer)

# 4 Supporting Molecular Characterization

^1^H-NMR spectrum of **CM** in DMSO-d_6_.


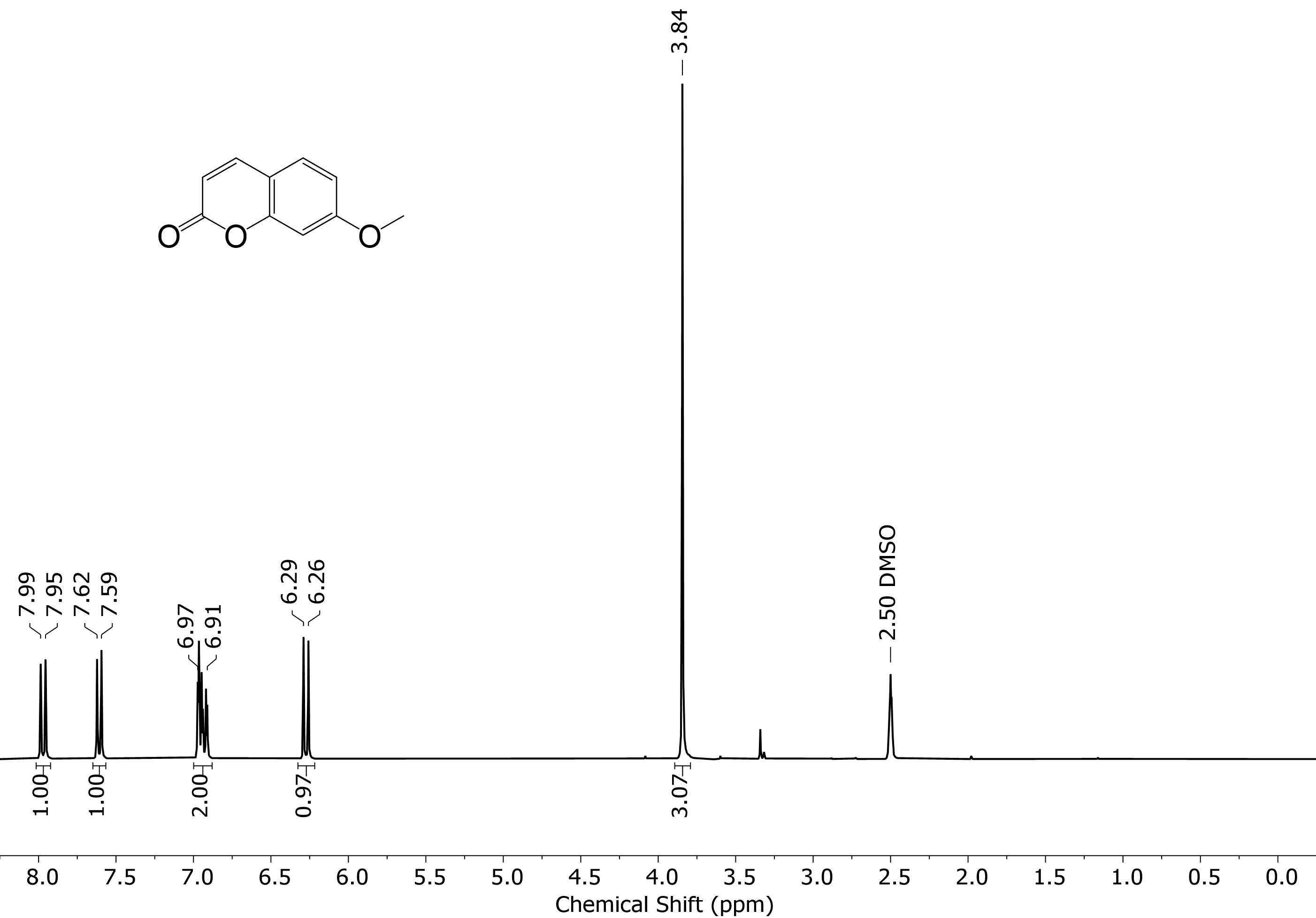


^13^C-NMR spectrum of **CM** in DMSO-d_6_.


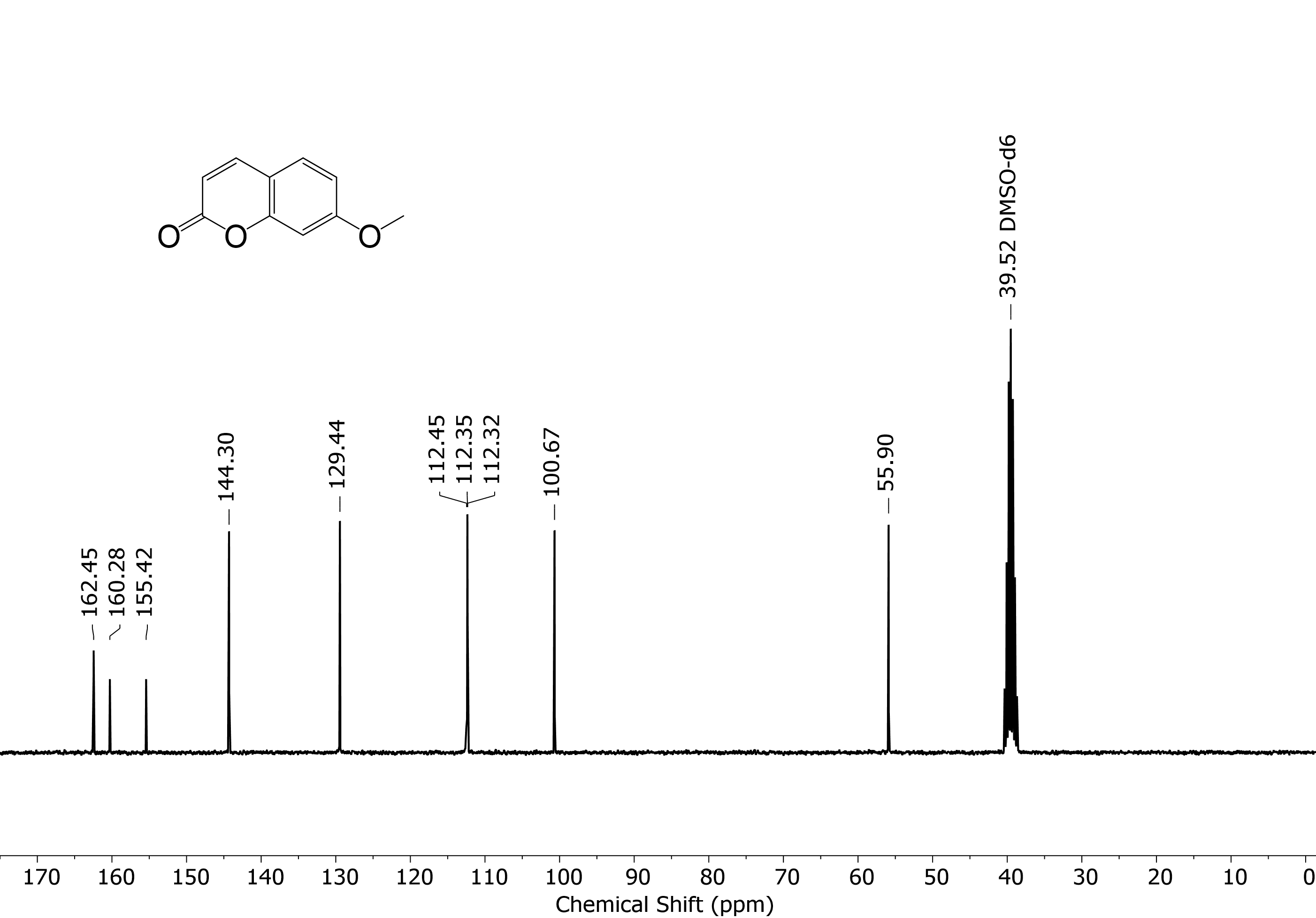


^1^H-NMR spectrum of a mixture of **CD*_sHT_*** and **CD*_sHH_*** in DMSO-d_6_.


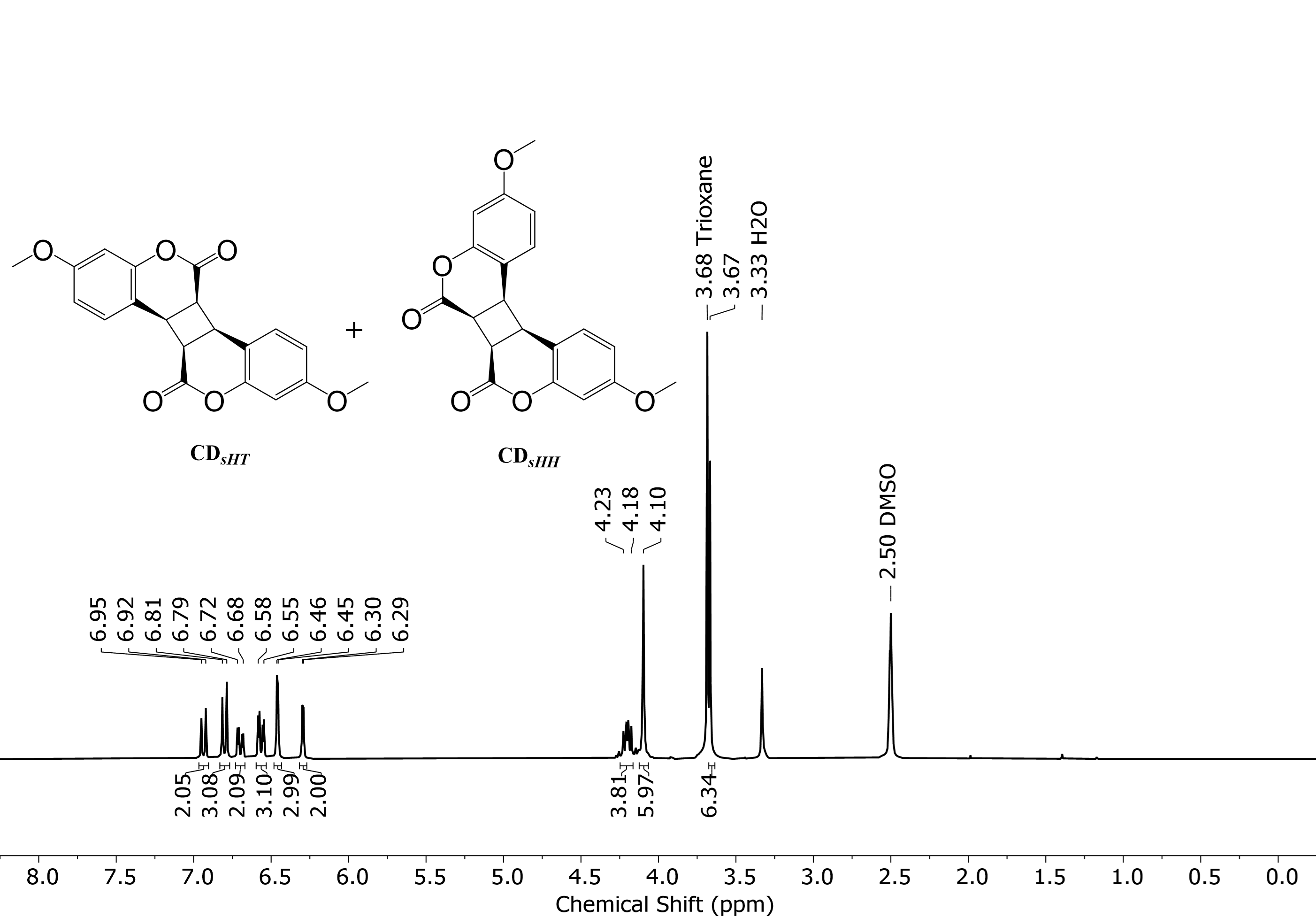


^13^C-NMR spectrum of a mixture of **CD*_sHT_*** and **CD*_sHH_*** in DMSO-d_6_.


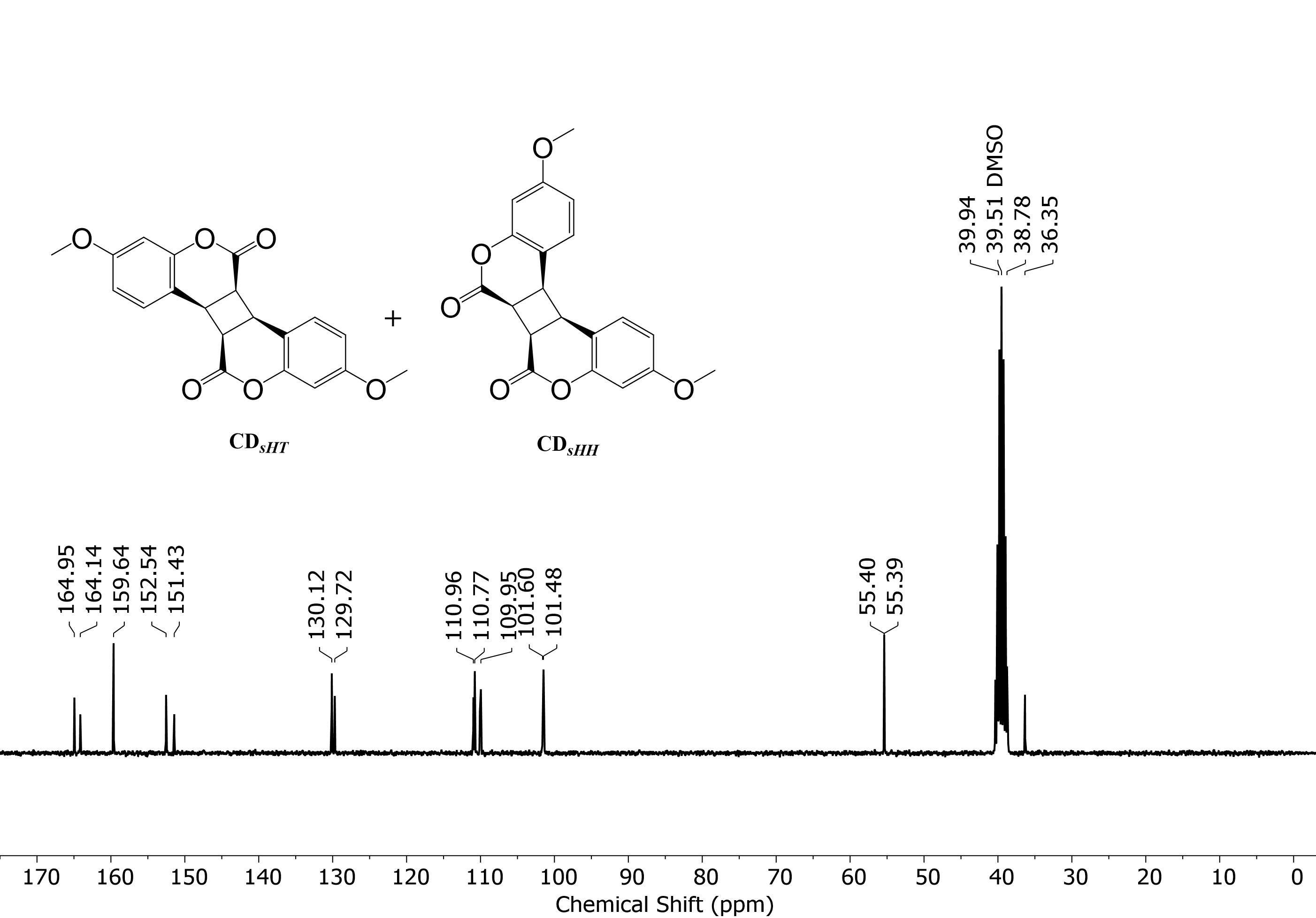


^1^H-NMR spectrum of **QM** in CDCl_3_.


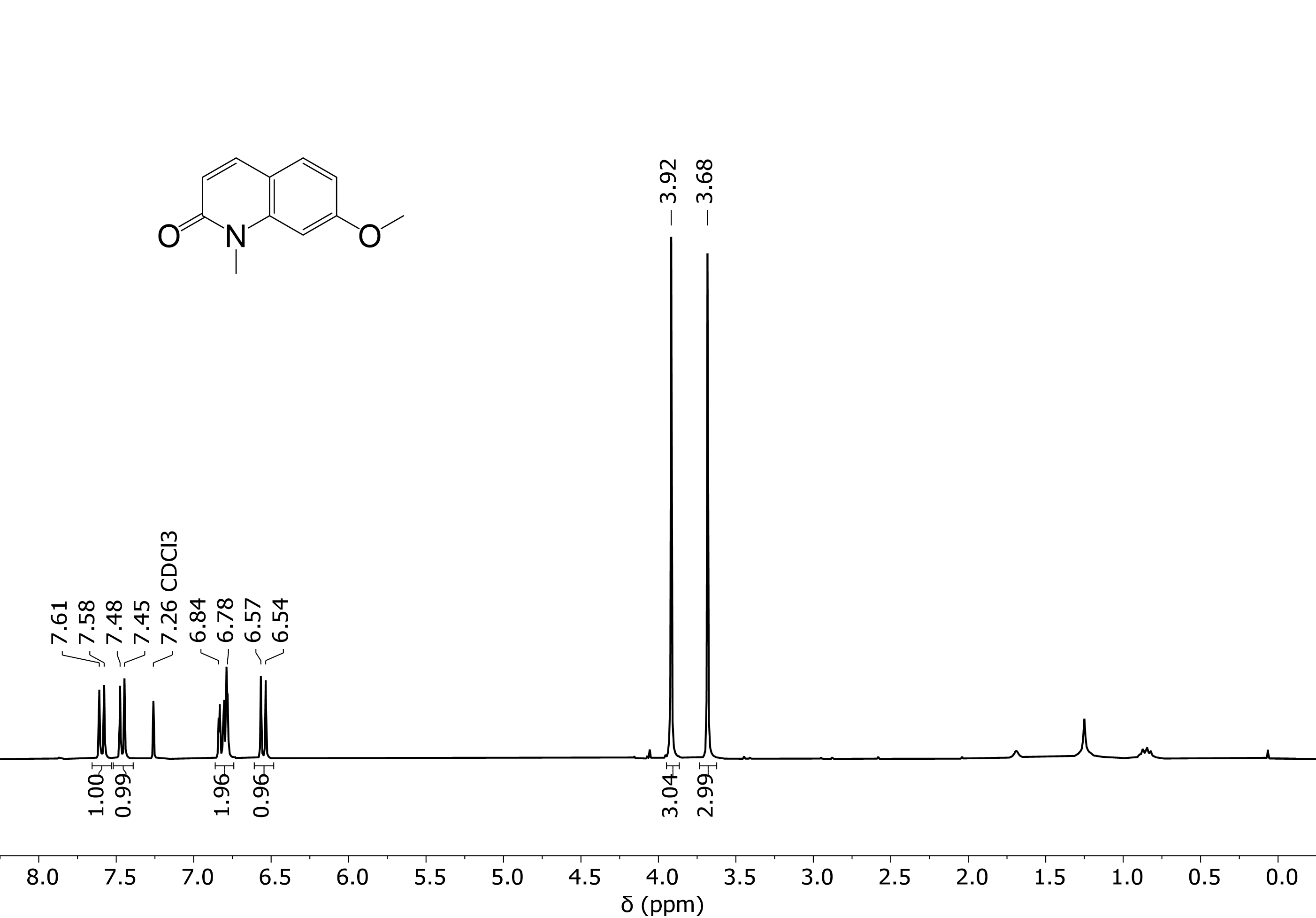


^13^C-NMR spectrum of **QM** in CDCl_3_.


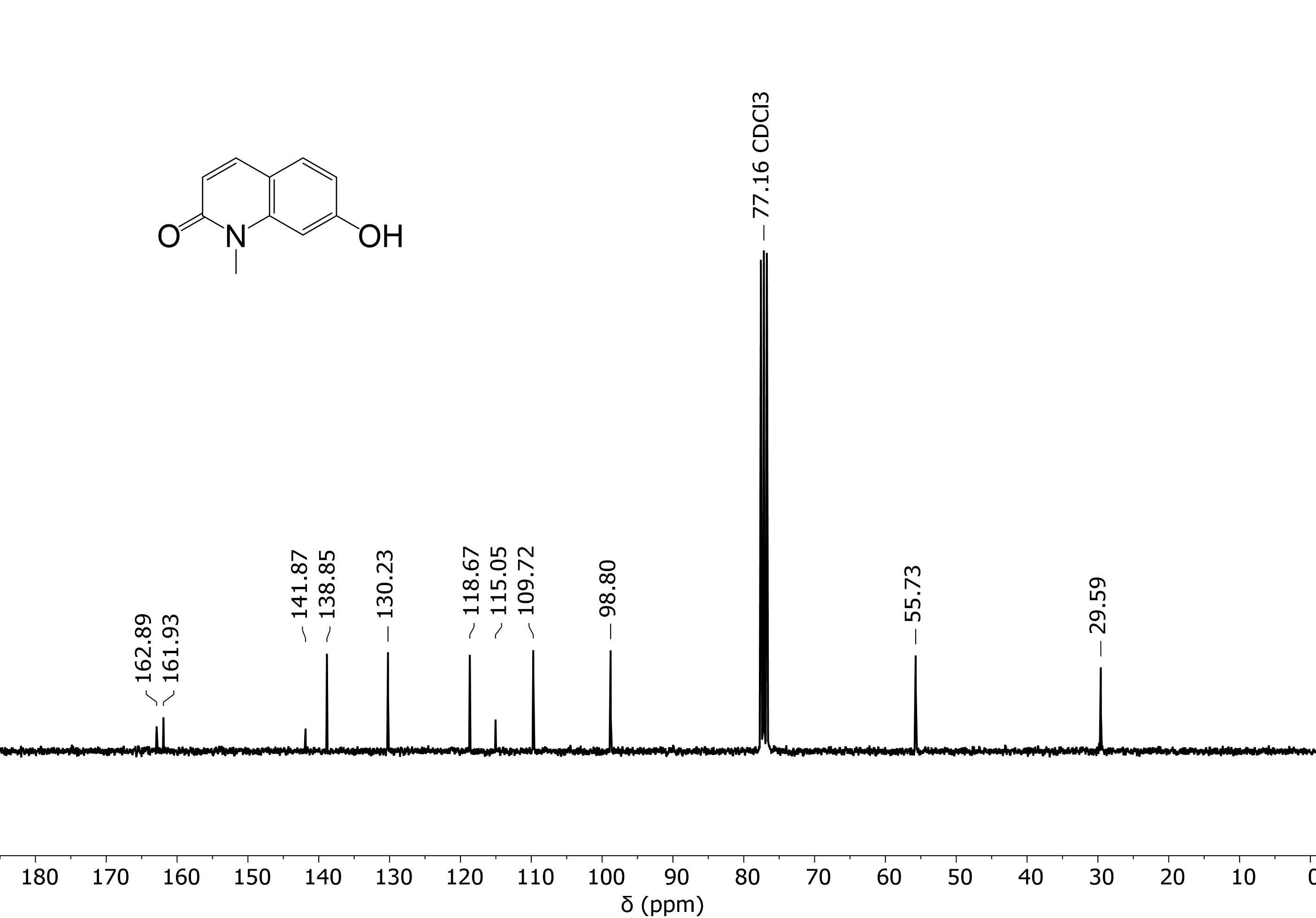


^1^H-NMR spectrum of **QD*_aHH_*** in CDCl_3_.


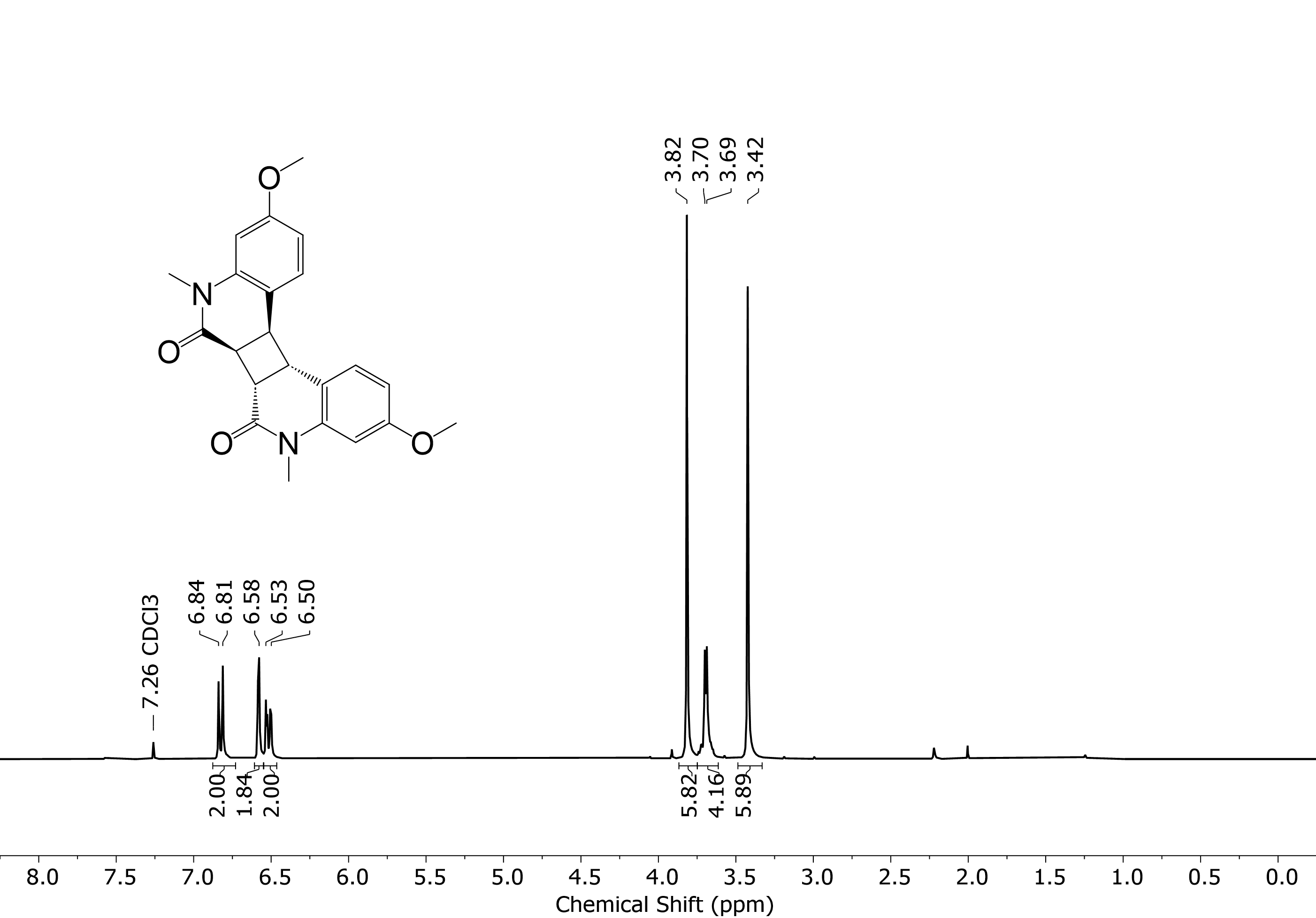


^13^C-NMR spectrum of **QD*_aHH_*** in CDCl_3_.


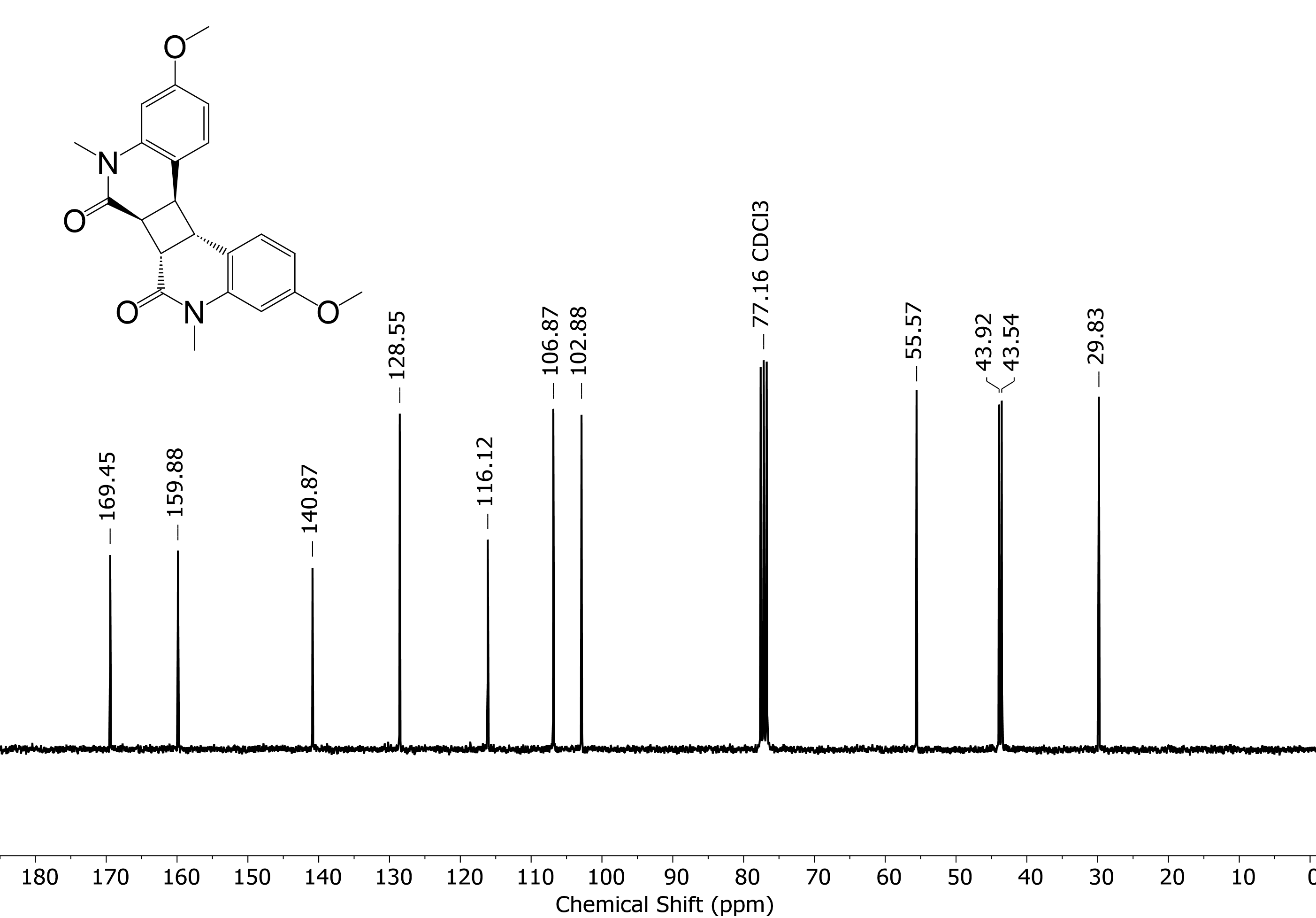


ESI spectrum of **QD*_aHH_***.


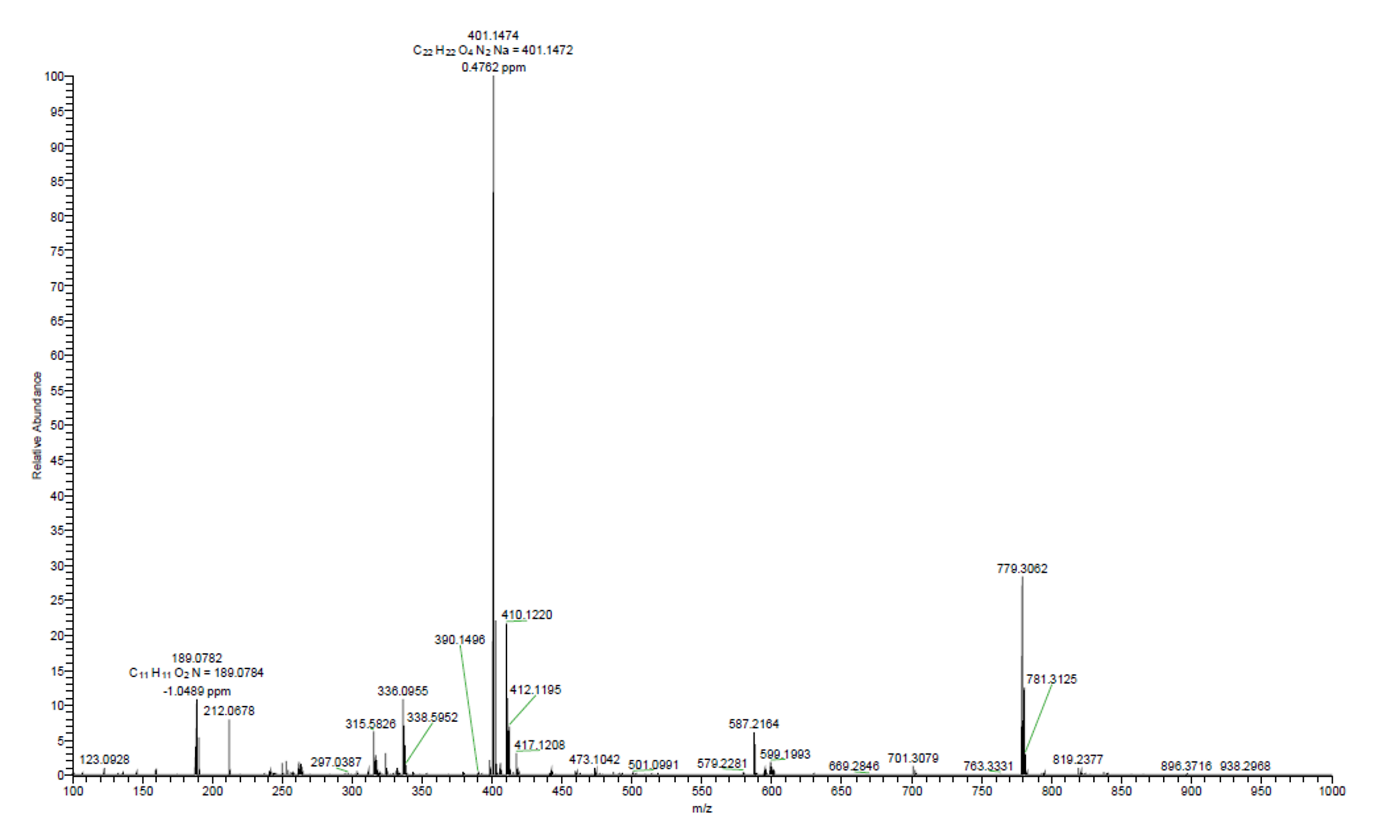


^1^H-NMR spectrum of **1** in DMSO-d_6_.


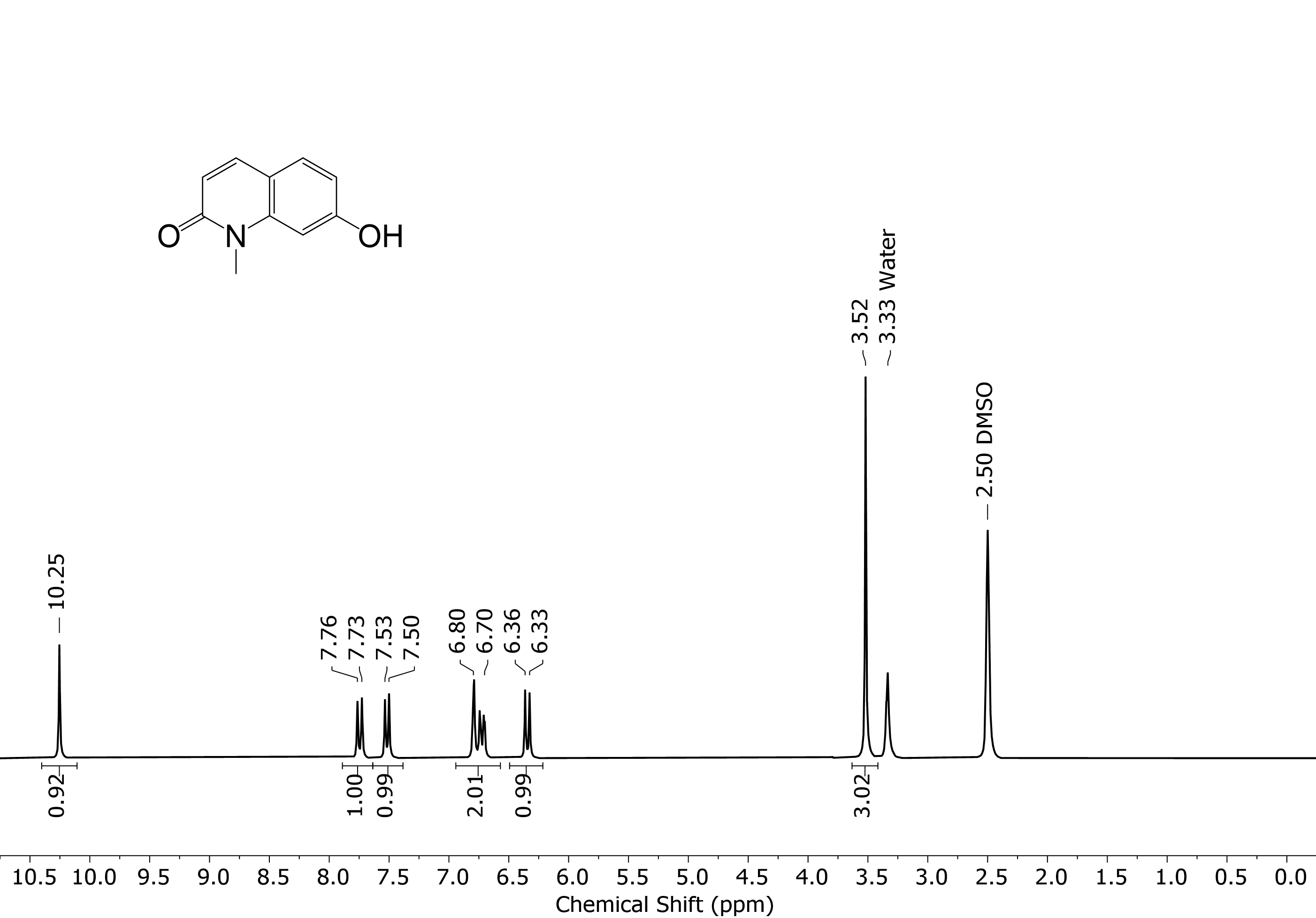


^13^C-NMR spectrum of **1** in DMSO-d_6_.


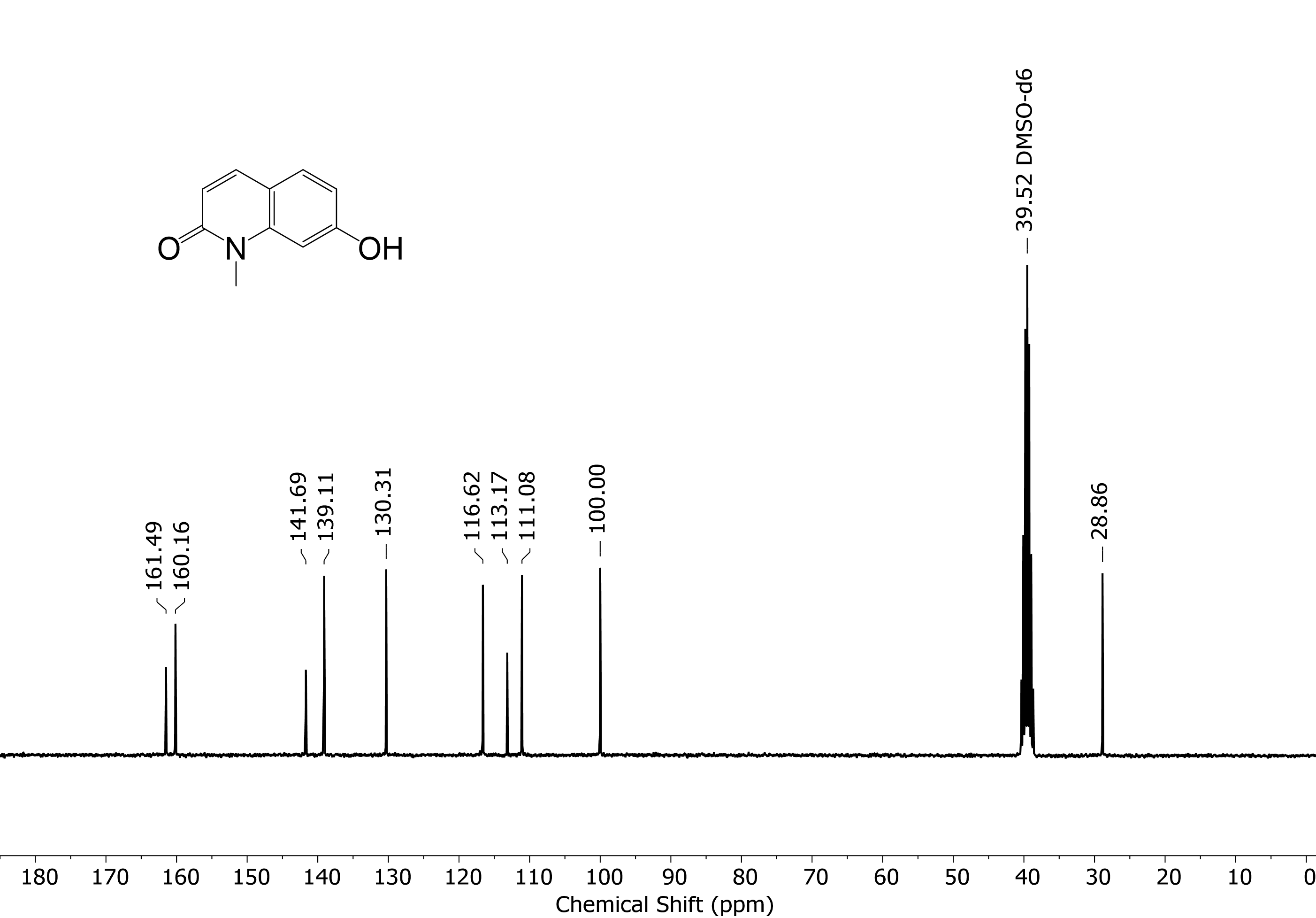


^1^H-NMR spectrum of **2** in DMSO-d_6_.

^
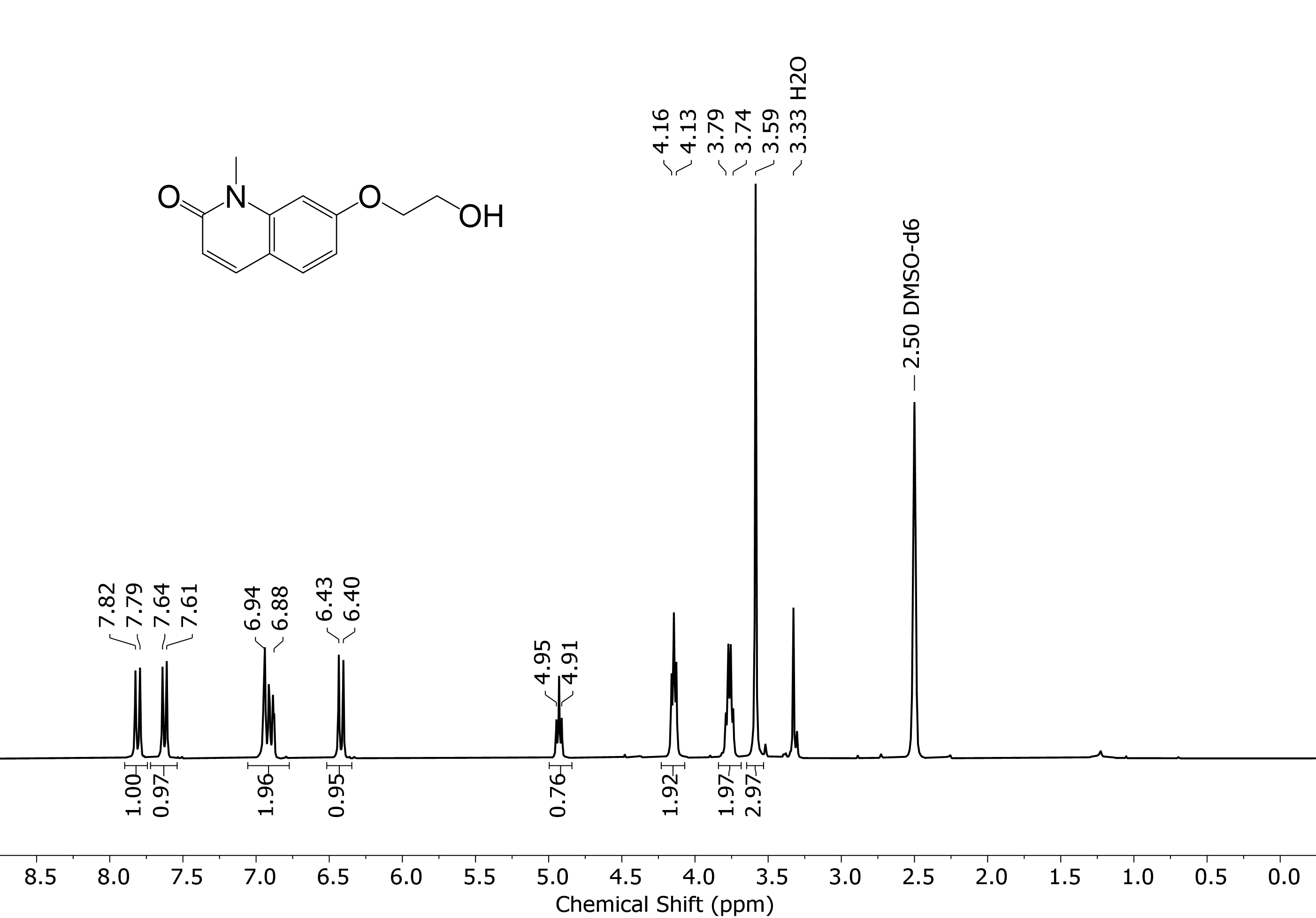
^

^13^C-NMR spectrum of **2** in DMSO-d_6_.

^
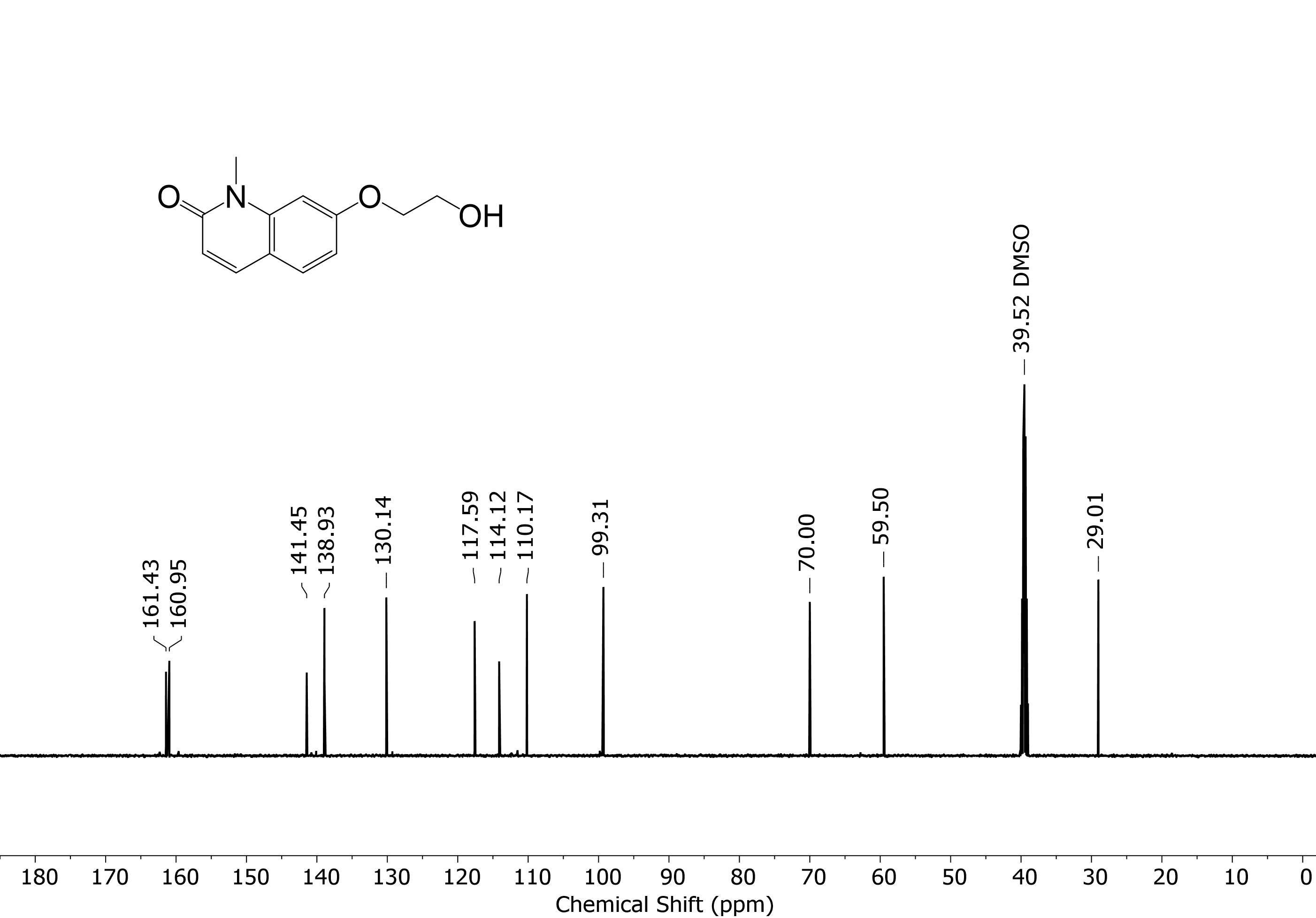
^

ESI spectrum of **(2)**.


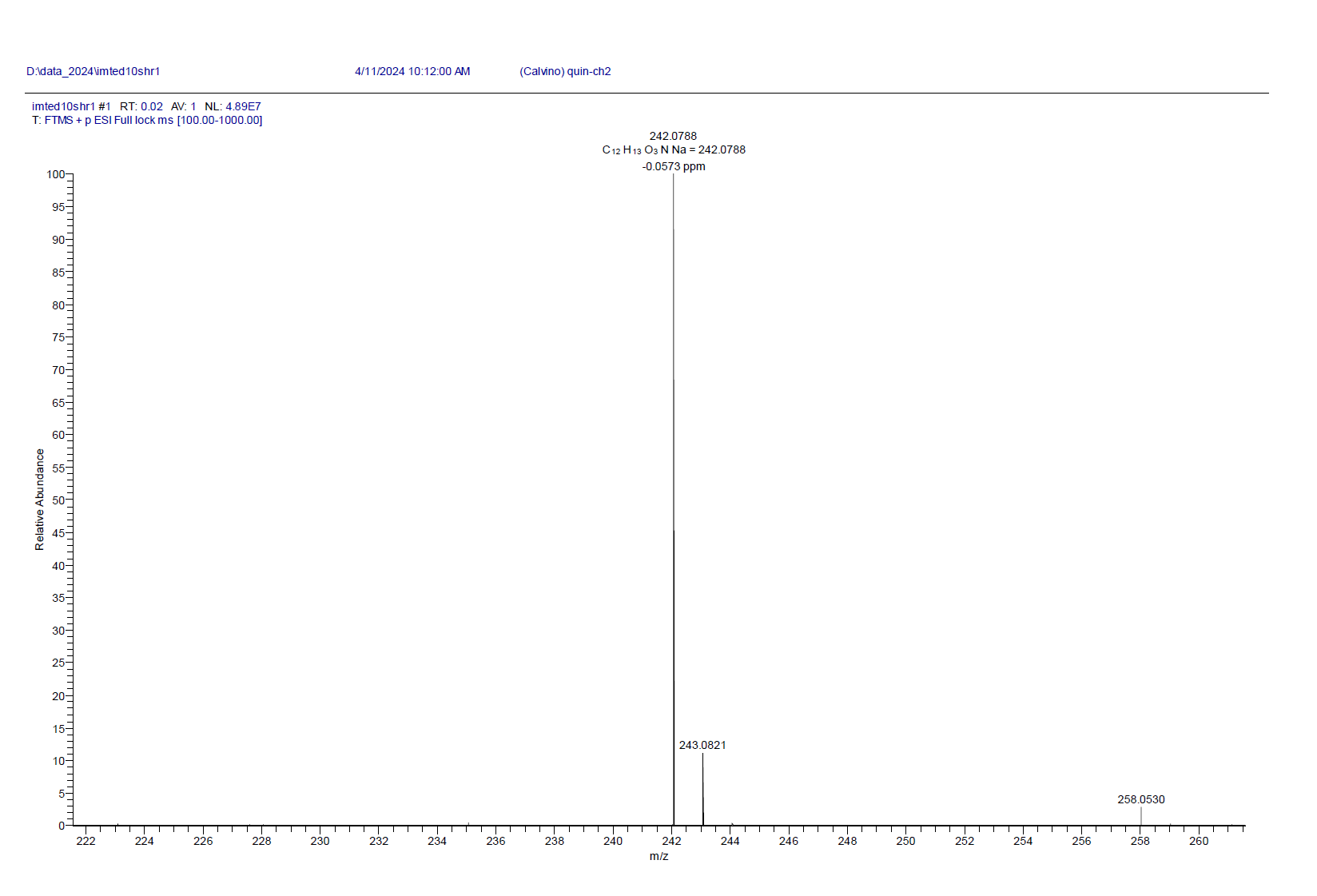


^1^H-NMR of **3** in DMSO-d_6._

_s
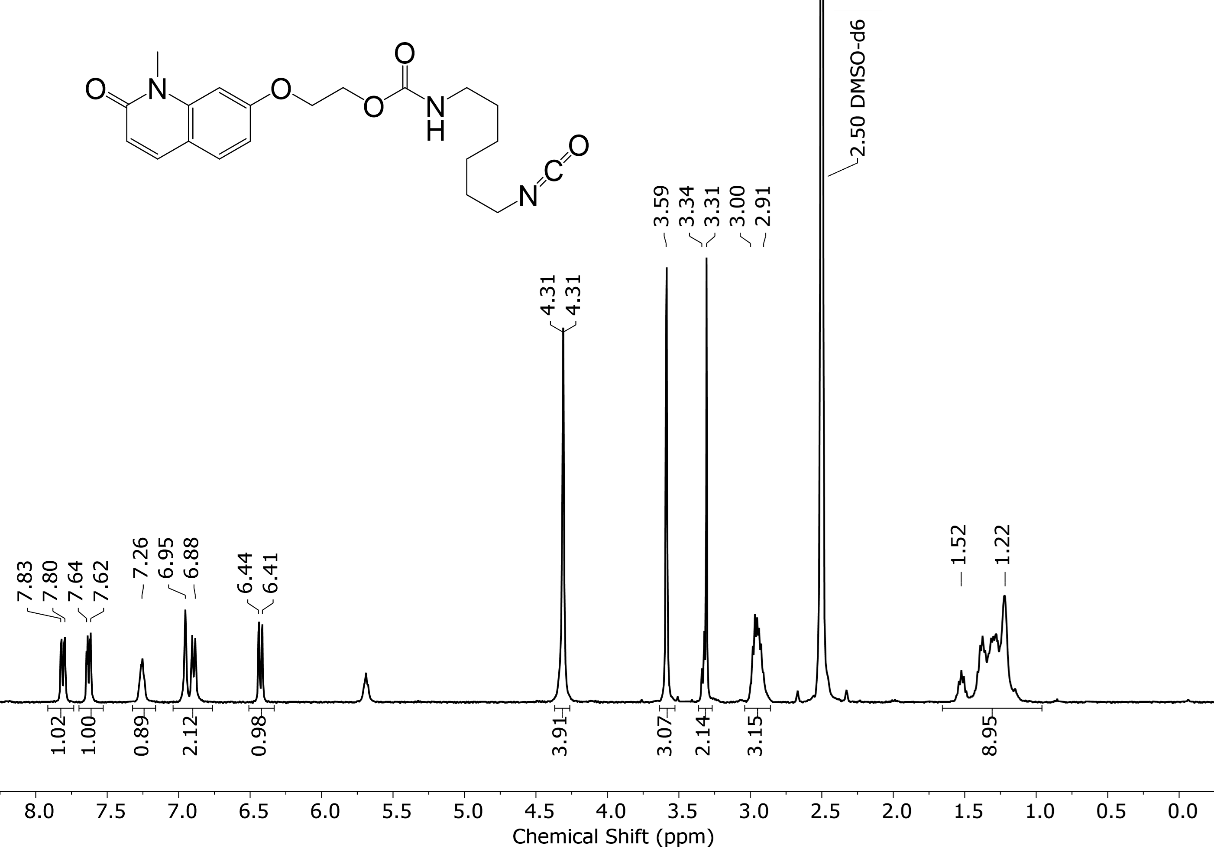
_

^1^H-NMR of **3** in DMSO-d_6._

_
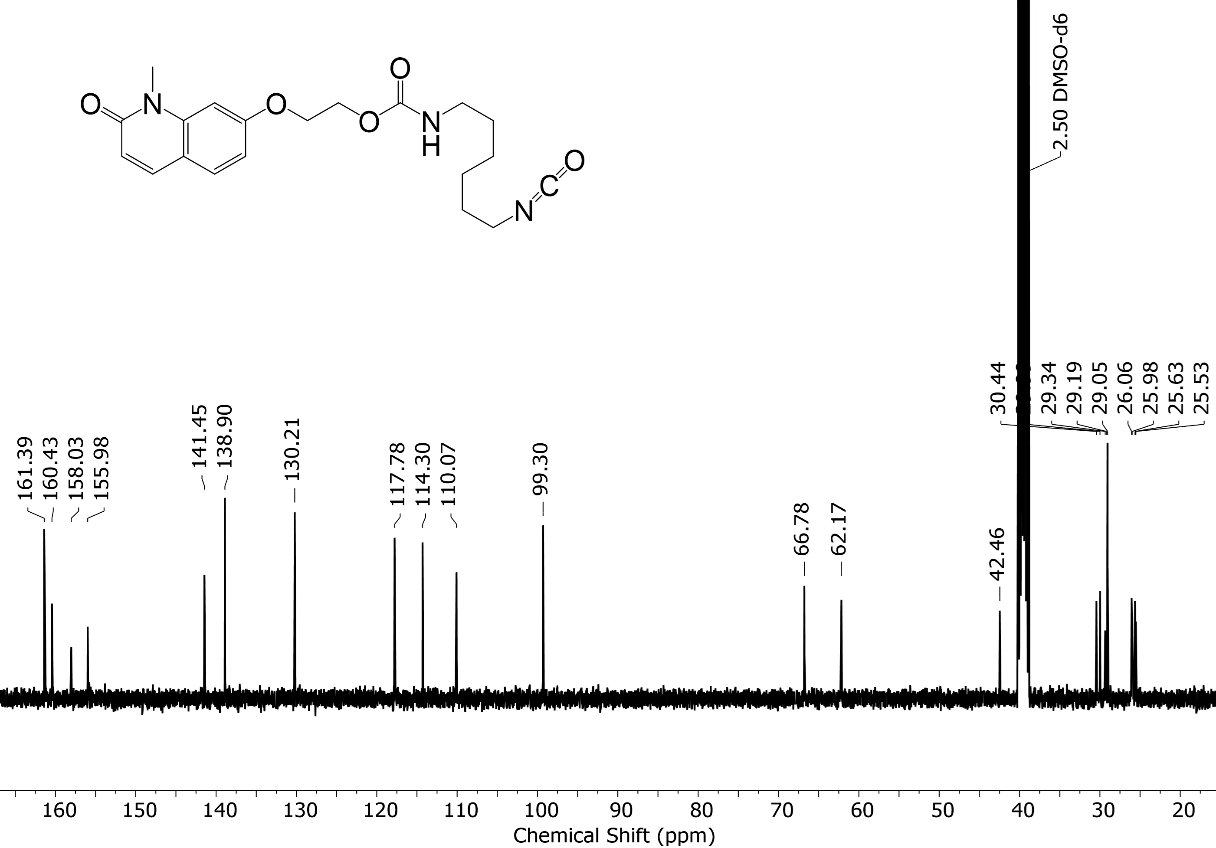
_

^1^H-NMR Spectrum of **PEG-QM** in DMSO-d_6._


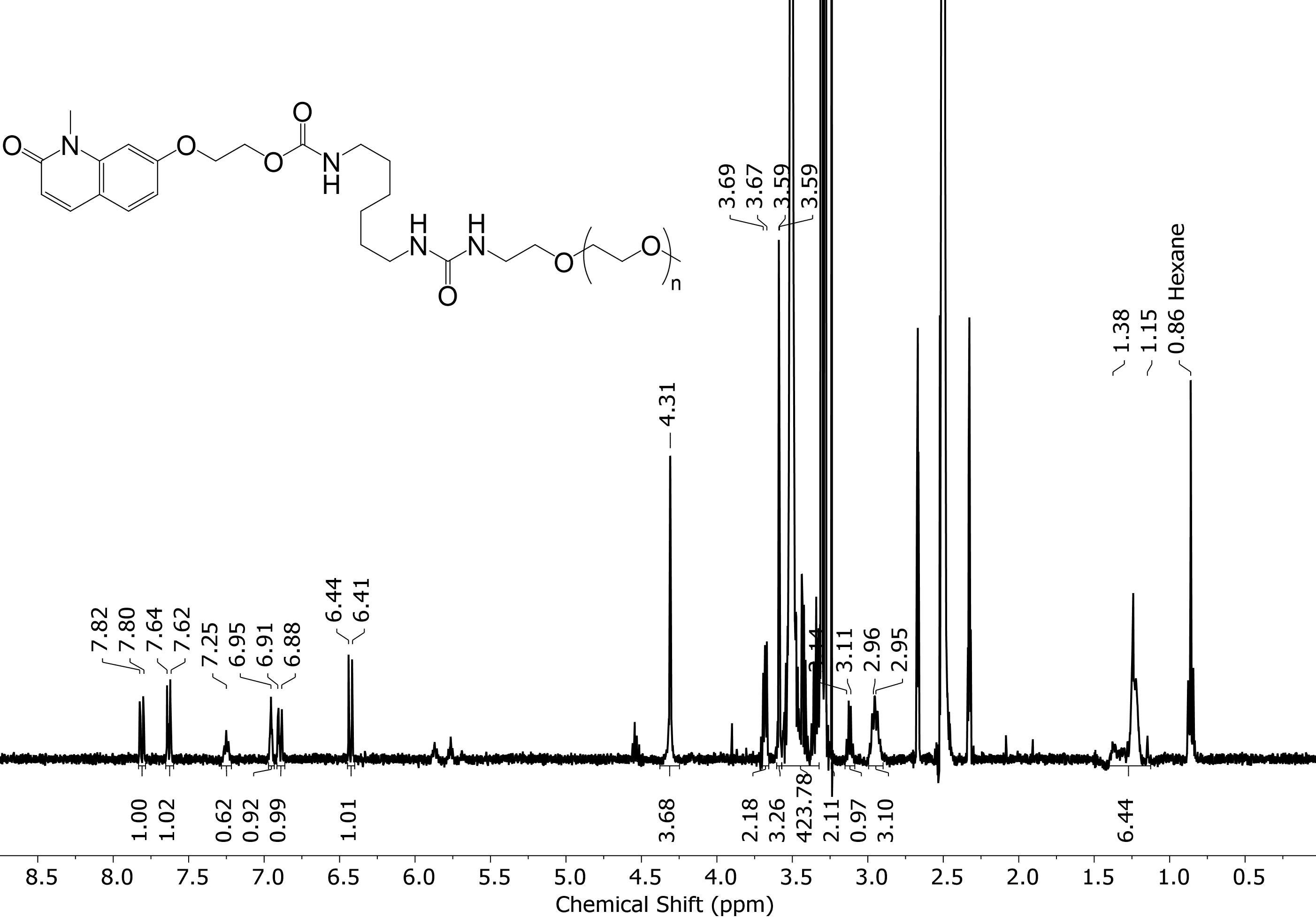


# 5 References

[1] Bruker (2012). SAINT, Bruker AXS Inc. Madison, WI, USA.

[2] L. Krause, R. Herbst-Irmer, G. M. Sheldrick, D. Stalke, *J. Appl. Crystallogr.* **2015**, *48*, 3–10.

[3] G. M. Sheldrick, *Acta Crystallogr. Sect. A Found. Adv.* **2015**, *71*, 3–8.

[4] C. R. Groom, I. J. Bruno, M. P. Lightfoot, S. C. Ward, *Acta Crystallogr. Sect. B Struct. Sci. Cryst. Eng. Mater.* **2016**, *72*, 171–179.

[5] Kratzer, D. FinalCif. GitHub repository. Available online: https://github.com/dkratzert/FinalCif (accessed June 20, 2024).

[6] G. R. Fulmer, A. J. M. Miller, N. H. Sherden, H. E. Gottlieb, A. Nudelman, B. M. Stoltz, J. E. Bercaw, K. I. Goldberg, *Organometallics* **2010**, *29*, 2176–2179.

[7] N. Paul, M. Jiang, N. Bieniek, J. L. P. Lustres, Y. Li, N. Wollscheid, T. Buckup, A. Dreuw, N. Hampp, M. Motzkus, *J. Phys. Chem. A* **2018**, *122*, 7587–7597.
